# Supplementary material for: Systematic review and meta-analysis for a Global Patient co-Owned Cloud (GPOC)
Source: Nat Commun. 2024 Mar 11;15:2186. doi: 10.1038/s41467-024-46503-5 (PMC10928077; doi:10.1038/s41467-024-46503-5)

SUPPLEMENTARY INFORMATION

Supplementary files appear in this document in the same order as they are referred to in the article text.

Document	Page
S1; Documentation of Search Strategies	**2-17**
S2; GPOC Supplementary Meta-Analysis Forest Plots	**18-36**
S3; PRISMA 2020 Checklist GPOC	**37-39**
S4; GPOC Word Cloud	**40**

S1; Documentation of Search Strategies

Documentation of search strategies
University Library search consultation group

Date: April 2022

Topic/research question: What is the global impact of the electronic health record on the patient and health care professional?

Name of researcher(s): Niklas Lidströmer, Women´s and Children´s Health

Librarian(s): Narcisa Hannerz & Anja Vikingson

Databases:

1. Medline (Ovid)
2. Embase (embase.com)
3. Cochrane Library (Wiley)
4. Web of Science (Clarivate analytics)
5. Cinahl (EBSCO)
6. Scopus (Elsevier)
7. Engineering Village (Via Imperial)

Total number of hits:

- Before deduplication: 16,045
- After deduplication: 9,362

Comments:

Deduplication based on the method described in:
Bramer, W. M., Giustini, D., de Jonge, G. B., Holland, L., & Bekhuis, T. (2016). De-duplication of database search results for systematic reviews in EndNote. *Journal of the Medical Library Association: JMLA*, 104(3), 240–243. doi:10.3163/1536-5050.104.3.014

One final, extra step was added to compare DOIs.

PRISMA 2020 flow diagram for new systematic reviews which included searches of databases and registers only[1]

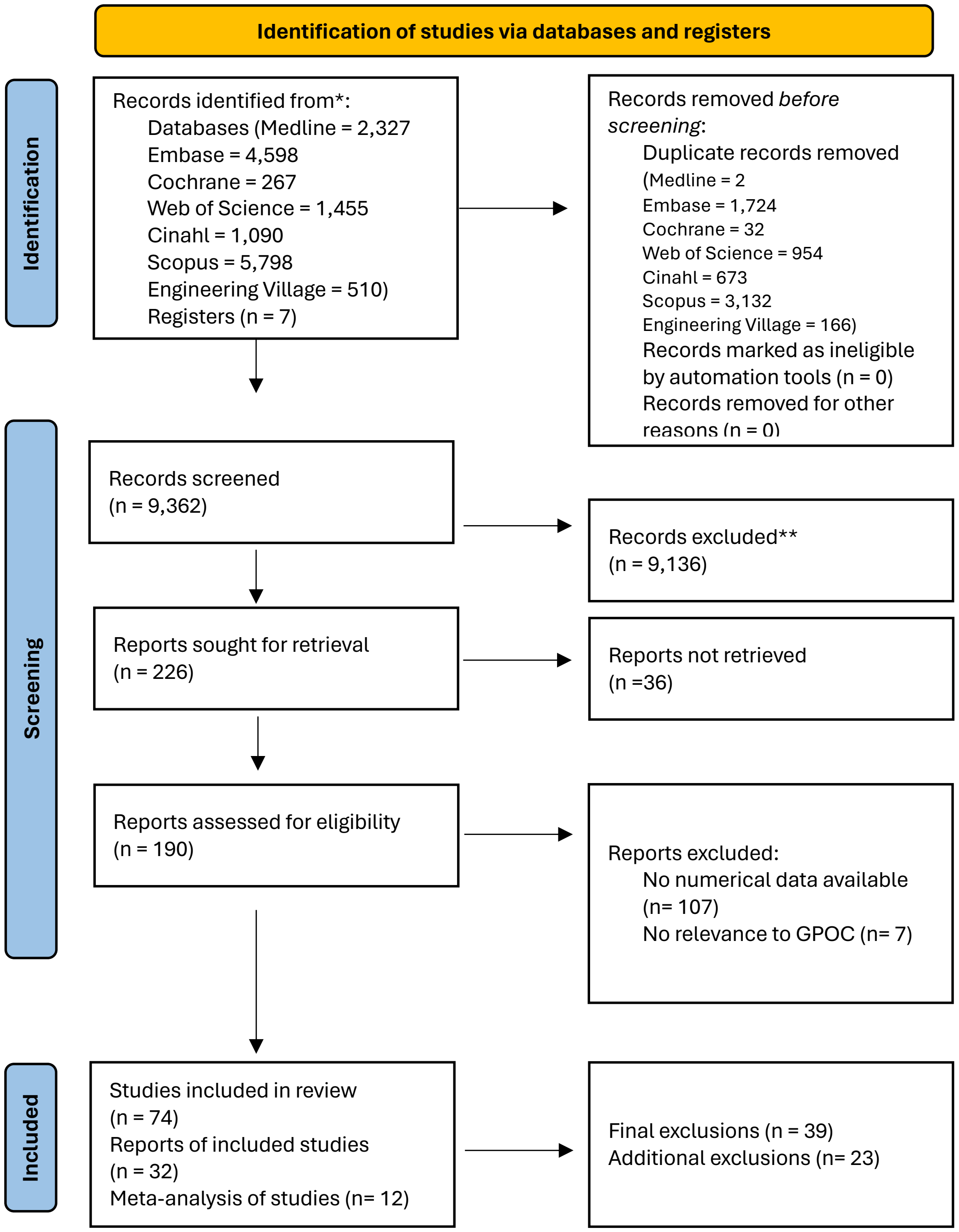

*Consider, if feasible to do so, reporting the number of records identified from each database or register searched (rather than the total number across all databases/registers).
**If automation tools were used, indicate how many records were excluded by a human and how many were excluded by automation tools.

From: Page MJ, McKenzie JE, Bossuyt PM, Boutron I, Hoffmann TC, Mulrow CD, et al. The PRISMA 2020 statement: an updated guideline for reporting systematic reviews. BMJ 2021;372:n71. doi: 10.1136/bmj.n71
For more information, visit: http://www.prisma-statement.org/

1. Different templates are available depending on the type of review (new or updated) and sources used to identify studies. For more information visit: http://prisma-statement.org/prismastatement/flowdiagram.aspx

1. Medline

Interface: Ovid MEDLINE(R) and Epub Ahead of Print, In-Process & Other Non-Indexed Citations and Daily<br><br>Date of Search: 20 April 2022<br><br>Number of hits: 2,327<br><br>Comment: In Ovid, two or more words are automatically searched as phrases; i.e. no quotation marks are needed	Field labels<br><br>• exp/ = exploded MeSH term<br>• / = non exploded MeSH term<br>• .ti,ab,kf. = title, abstract and author keywords<br>• adjx = within x words, regardless of order<br>• * = truncation of word for alternate endings

Database(s): **Ovid MEDLINE(R) and Epub Ahead of Print, In-Process, In-Data-Review & Other Non-Indexed Citations and Daily** 1946 to April 19, 2022
Search Strategy:

#	Searches
1	Decision Support Systems, Clinical/
2	Decision Support Systems, Management/
3	exp Electronic Health Records/
4	Health Information Systems/
5	Health Records, Personal/
6	Medical Record Linkage/
7	Medical Records Systems, Computerized/
8	Patient Portals/
9	(ehr or ehrs or emr or emrs).ti,ab.
10	((computeri#ed or electronic or personal or personali#ed) adj2 (health* data* or health* record* or medical record* or patient record* or pharmaceutical record*)).ti,ab,kf.
11	(system* adj2 (clinical decision or clinical information or decision support or health information or medical information)).ti,ab,kf.
12	(cds system* or medical record linkage* or patient* portal* or personal health information).ti,ab,kf.
13	or/1-12
14	exp Health Personnel/
15	Patient Participation/
16	exp Patients/
17	(client? or hospitaliz* or inpatient* or in-patient* or institution* or outpatient* or out-patient or patient*).ti,ab,kf.
18	((clinical or health* or medical or nurse or nursing) adj3 (organization* or personnel or professional* or provider* or staff)).ti,ab,kf.
19	(allergist? or anesthesiologist? or anaesthesiologist? or cardiologist? or dermatologist? or endocrinologist? or gastroenterologist? or general practitioner? or geriatrician? or midwife* or midwive* or neonatologist? or nephrologist? or neurologist? or nurse* or oncologist? or ophthalmologist? or otolaryngologist? or pathologist? or paediatrician? or pediatrician? or pharmacist* or physiatrist? or physician? or physical therapist or physiotherapist? or physio-therapist? or psychotherapist? or psycho-therapist? or pulmonologist? or radiologist? or rheumatologist? or surgeon? or urologist?).ti,ab,kf.
20	or/14-19

21	Artificial Intelligence/
22	Cloud Computing/
23	Data Management/
24	Information Dissemination/
25	"Information Storage and Retrieval"/
26	exp Machine Learning/
27	Natural Language Processing/
28	((artificial or comput* or machine) adj2 intelligence).ti,ab,kf.
29	(computer adj2 (reasoning or vision system)).ti,ab,kf.
30	((data or information) adj3 (administ* or custod* or dissemination or distribution or extract* or govern* or link* or manage* or retrieval* or source* or sharing* or steward* or storage*)).ti,ab,kf.
31	(learning adj3 (deep or hierarchical or label?ed data or machine or semi-supervised or transfer)).ti,ab,kf.
32	((machine? or network?) adj2 support vector).ti,ab,kf.
33	(cloud* or knowledge acquisition or knowledge representation? or natural language process*).ti,ab,kf.
34	or/21-33
35	exp Computer Security/
36	Confidentiality/
37	Ownership/
38	Patient Access to Records/
39	Patient Rights/
40	"legislation & jurisprudence".fs.
41	(anonymi#ation or blockchain* or block chain* or civil suit* or confidential* or co-own* or coown* or cybersecurit* or de-Identification? or deIdentification? or electronic signature* or encrypt* or jurisprudence or law* or legal or legislat* or ownership* or privileged communication or regulation* or secrecy or secur*).ti,ab,kf.
42	((computer or cyber or data) adj3 (breach* or compromising or hacker? or virus or viruses or worm*)).ti,ab,kf.
43	((data or information) adj3 (masking or protection)).ti,ab,kf.
44	((patient* or property) adj2 right*).ti,ab,kf.
45	((patient or data) adj2 privacy).ti,ab,kf.
46	or/35-45
47	13 and 20 and 34 and 46

2. Embase

Interface: embase.com<br><br>Date of Search: 20 April 2022<br><br>Number of hits: 4,598<br><br>Comment: Emtree is the controlled vocabulary in Embase	Field labels<br><br>• /exp = exploded Emtree term<br>• /de = non exploded Emtree term<br>• ti,ab,kw = title, abstract and author keywords<br>• NEAR/x = within x words, regardless of order<br>• * = truncation of word for alternate endings

#53	#13 AND #20 AND #38 AND #52
#52	#39 OR #40 OR #41 OR #42 OR #43 OR #44 OR #45 OR #46 OR #47 OR #48 OR #49 OR #50 OR #51
#51	((patient OR data) NEAR/2 privacy):ti,ab,kw
#50	((patient* OR property) NEAR/2 right*):ti,ab,kw
#49	((data OR information) NEAR/3 (masking OR protection)):ti,ab,kw
#48	((computer OR cyber OR data) NEAR/3 (breach* OR compromising OR hacker$ OR virus OR viruses OR worm*)):ti,ab,kw
#47	anonymi?ation:ti,ab,kw OR blockchain*:ti,ab,kw OR 'block chain*':ti,ab,kw OR 'civil suit*':ti,ab,kw OR confidential*:ti,ab,kw OR 'co-own*':ti,ab,kw OR coown*:ti,ab,kw OR cybersecurit*:ti,ab,kw OR 'de-identification$':ti,ab,kw OR deidentification$:ti,ab,kw OR 'electronic signature*':ti,ab,kw OR encrypt*:ti,ab,kw OR jurisprudence:ti,ab,kw OR law*:ti,ab,kw OR legal:ti,ab,kw OR legislat*:ti,ab,kw OR ownership*:ti,ab,kw OR 'privileged communication':ti,ab,kw OR regulation*:ti,ab,kw OR secrecy:ti,ab,kw OR secur*:ti,ab,kw
#46	'patient right'/de
#45	'legal aspect'/de
#44	'law'/de
#43	'jurisprudence'/de
#42	'information security'/de
#41	'data protection'/exp
#40	'confidentiality'/exp
#39	'computer security'/exp
#38	#21 OR #22 OR #23 OR #24 OR #25 OR #26 OR #27 OR #28 OR #29 OR #30 OR #31 OR #32 OR #33 OR #34 OR #35 OR #36 OR #37
#37	cloud*:ti,ab,kw OR 'knowledge acquisition':ti,ab,kw OR 'knowledge representation$':ti,ab,kw OR 'natural language process*':ti,ab,kw
#36	((machine$ OR network$) NEAR/2 'support vector'):ti,ab,kw
#35	(learning NEAR/3 (deep OR hierarchical OR 'label$ed data' OR machine OR 'semi supervised' OR transfer)):ti,ab,kw
#34	((data OR information) NEAR/3 (administ* OR custod* OR dissemination OR distribution OR extract* OR govern* OR link* OR manage* OR retrieval* OR source* OR sharing* OR steward* OR storage*)):ti,ab,kw
#33	(computer NEAR/2 (reasoning OR 'vision system')):ti,ab,kw
#32	((artificial OR comput* OR machine) NEAR/2 intelligence):ti,ab,kw
#31	'natural language processing'/de
#30	'deep learning'/de
#29	'support vector machine'/exp

#28	'unsupervised machine learning'/de
#27	'supervised machine learning'/de
#26	'machine learning'/de
#25	'information storage'/de
#24	'information retrieval'/de
#23	'information processing'/de
#22	'cloud computing'/de
#21	'artificial intelligence'/de
#20	#14 OR #15 OR #16 OR #17 OR #18 OR #19
#19	allergist$:ti,ab,kw OR anesthesiologist$:ti,ab,kw OR anaesthesiologist$:ti,ab,kw OR cardiologist$:ti,ab,kw OR dermatologist$:ti,ab,kw OR endocrinologist$:ti,ab,kw OR gastroenterologist$:ti,ab,kw OR 'general practitioner$':ti,ab,kw OR geriatrician$:ti,ab,kw OR midwife*:ti,ab,kw OR midwive*:ti,ab,kw OR neonatologist$:ti,ab,kw OR nephrologist$:ti,ab,kw OR neurologist$:ti,ab,kw OR nurse*:ti,ab,kw OR oncologist$:ti,ab,kw OR ophthalmologist$:ti,ab,kw OR otolaryngologist$:ti,ab,kw OR pathologist$:ti,ab,kw OR pediatrician$:ti,ab,kw OR paediatrician$:ti,ab,kw OR pharmacist*:ti,ab,kw OR physiatrist$:ti,ab,kw OR physician$:ti,ab,kw OR 'physical therapist$':ti,ab,kw OR physiotherapist$:ti,ab,kw OR 'physio-therapist$':ti,ab,kw OR psychotherapist$:ti,ab,kw OR 'psycho-therapist$':ti,ab,kw OR pulmonologist$:ti,ab,kw OR radiologist$:ti,ab,kw OR rheumatologist$:ti,ab,kw OR surgeon$:ti,ab,kw OR urologist$:ti,ab,kw
#18	((clinical OR health* OR medical OR nurse OR nursing) NEAR/3 (organi?ation* OR personnel OR professional* OR provider* OR staff)):ti,ab,kw
#17	client$:ti,ab,kw OR hospitali*:ti,ab,kw OR inpatient*:ti,ab,kw OR institution*:ti,ab,kw OR outpatient*:ti,ab,kw OR patient*:ti,ab,kw
#16	'patient'/exp
#15	'patient participation'/de
#14	'health care personnel'/exp
#13	#1 OR #2 OR #3 OR #4 OR #5 OR #6 OR #7 OR #8 OR #9 OR #10 OR #11 OR #12
#12	'cds system*':ti,ab,kw OR 'medical record linkage*':ti,ab,kw OR 'patient* portal*':ti,ab,kw OR 'personal health information':ti,ab,kw
#11	(system* NEAR/2 ('clinical decision' OR 'clinical information' OR 'decision support' OR 'health information' OR 'medical information')):ti,ab,kw
#10	((computeri?ed OR electronic OR personal OR personali?ed) NEAR/2 ('health* data*' OR 'health* record*' OR 'medical record*' OR 'patient record*' OR 'pharmaceutical record*')):ti,ab,kw
#9	ehr:ti,ab OR ehrs:ti,ab OR emr:ti,ab OR emrs:ti,ab
#8	'electronic medical record system'/de
#7	'medical record'/de
#6	'medical information system'/de
#5	'electronic patient record'/de
#4	'electronic medical record'/de
#3	'electronic health record'/de
#2	'decision support system'/de
#1	'clinical decision support system'/de

3. Cochrane Library

Interface: Wiley<br><br>Date of Search: 20 April 2022<br><br>Number of hits: 267	Field labels<br><br>• ti,ab,kw = title, abstract and author keywords<br>• NEAR/x = within x words, regardless of order<br>• * = truncation of word for alternate endings

#1	MeSH descriptor: [Decision Support Systems, Clinical] this term only
#2	MeSH descriptor: [Decision Support Systems, Management] this term only
#3	MeSH descriptor: [Electronic Health Records] explode all trees
#4	MeSH descriptor: [Health Information Systems] this term only
#5	MeSH descriptor: [Health Records, Personal] this term only
#6	MeSH descriptor: [Medical Record Linkage] this term only
#7	MeSH descriptor: [Medical Records Systems, Computerized] this term only
#8	MeSH descriptor: [Patient Portals] this term only
#9	(ehr OR ehrs OR emr OR emrs):ti,ab
#10	((computeri?ed OR electronic OR personal OR personali?ed) NEAR/2 ((health* NEXT data*) OR (health* NEXT record*) OR (medical NEXT record*) OR (patient NEXT record*) OR (pharmaceutical NEXT record*))):ti,ab,kw
#11	(system* NEAR/2 ("clinical decision" OR "clinical information" OR "decision support" OR "health information" OR "medical information")):ti,ab,kw
#12	((cds NEXT system*) OR ("medical record" NEXT linkage*) OR (patient* NEXT portal*) OR "personal health information"):ti,ab,kw
#13	#1 OR #2 OR #3 OR #4 OR #5 OR #6 OR #7 OR #8 OR #9 OR #10 OR #11 OR #12
#14	MeSH descriptor: [Health Personnel] explode all trees
#15	MeSH descriptor: [Patient Participation] this term only
#16	MeSH descriptor: [Patients] explode all trees
#17	(client* OR hospitali* OR inpatient* OR institution* OR outpatient* OR patient*):ti,ab,kw
#18	((clinical OR health* OR medical OR nurse OR nursing) NEAR/3 (organi?ation* OR personnel OR professional* OR provider* OR staff)):ti,ab,kw
#19	(allergist* OR anesthesiologist* OR anaesthesiologist* OR cardiologist* OR dermatologist* OR endocrinologist* OR gastroenterologist* OR "general practitioner*" OR geriatrician* OR midwife* OR midwive* OR neonatologist* OR nephrologist* OR neurologist* OR nurse* OR oncologist* OR ophthalmologist* OR otolaryngologist* OR pathologist* OR paediatrician* OR pediatrician* OR pharmacist* OR physiatrist* OR physician* OR "physical therapist*" OR physiotherapist* OR "physio-therapist*" OR psychotherapist* OR "psycho-therapist*" OR pulmonologist* OR radiologist* OR rheumatologist* OR surgeon* OR urologist*):ti,ab,kw
#20	#14 OR #15 OR #16 OR #17 OR #18 OR #19
#21	MeSH descriptor: [Artificial Intelligence] this term only
#22	MeSH descriptor: [Cloud Computing] this term only
#23	MeSH descriptor: [Data Management] this term only
#24	MeSH descriptor: [Information Dissemination] this term only
#25	MeSH descriptor: [] explode all trees
#26	MeSH descriptor: [Machine Learning] explode all trees
#27	MeSH descriptor: [Natural Language Processing] this term only
#28	((artificial OR comput* OR machine) NEAR/2 intelligence):ti,ab,kw
#29	(computer NEAR/2 (reasoning OR "vision system")):ti,ab,kw

#30	((data OR information) NEAR/3 (administ* OR custod* OR dissemination OR distribution OR extract* OR govern* OR link* OR manage* OR retrieval* OR source* OR sharing* OR steward* OR storage*)):ti,ab,kw
#31	(learning NEAR/3 (deep OR hierarchical OR "labeled data" OR "labelled data" OR machine OR "semi-supervised" OR transfer)):ti,ab,kw
#32	((machine* OR network*) NEAR/2 "support vector"):ti,ab,kw
#33	(cloud* OR (knowledge NEXT acquisition) OR (knowledge NEXT representation*) OR ("natural language" NEXT process*)):ti,ab,kw
#34	#21 OR #22 OR #23 OR #24 OR #25 OR #26 OR #27 OR #28 OR #29 OR 30 OR #31 OR #32 OR #33
#35	MeSH descriptor: [Computer Security] explode all trees
#36	MeSH descriptor: [Confidentiality] this term only
#37	MeSH descriptor: [Ownership] this term only
#38	MeSH descriptor: [Patient Access to Records] this term only
#39	MeSH descriptor: [Patient Rights] this term only
#40	MeSH descriptor: [] explode all trees and with qualifier(s): [legislation & jurisprudence - LJ]
#41	(anonymi?ation OR blockchain* OR (block NEXT chain*) OR (civil NEXT suit*) OR confidential* OR (co NEXT own*) OR coown* OR cybersecurit* OR (de NEXT identification*) OR deidentification* OR (electronic NEXT signature*) OR encrypt* OR jurisprudence OR law* OR legal OR legislat* OR ownership* OR "privileged communication" OR regulation* OR secrecy OR secur*):ti,ab,kw
#42	((computer OR cyber OR data) NEAR/3 (breach* OR compromising OR hacker* OR virus OR viruses OR worm*)):ti,ab,kw
#43	((data OR information) NEAR/3 (masking OR protection)):ti,ab,kw
#44	((patient* OR property) NEAR/2 right*):ti,ab,kw
#45	((patient OR data) NEAR/2 privacy):ti,ab,kw
#46	#35 OR #36 OR #37 OR #38 OR #39 OR #40 OR #41 OR #42 OR #43 OR #44 OR #45
#47	#13 AND #20 AND #34 AND #46
#48	#9 OR #10 OR #11 OR #12
#49	#17 OR #18 OR #19
#50	#28 OR #29 OR 30 OR #31 OR #32 OR #33
#51	#41 OR #42 OR #43 OR #44 OR #45
#52	#48 AND #49 AND #50 AND #51
#53	#47 NOT #52

4. Web of Science Core Collection

Interface: Clarivate Analytics<br><br>Date of Search: 20 April 2022<br><br>Number of hits: 1,455<br><br>Searched in: Editions = A&HCI , ESCI , SCI-EXPANDED , SSCI	Field labels<br><br>• TS/Topic = title, abstract, author keywords and Keywords Plus<br>• NEAR/x = within x words, regardless of order<br>• * = truncation of word for alternate endings<br><br>Note: sometimes “quotation marks” are needed for single search terms to avoid automatic term mapping (lemmatization).

1	TI=(ehr OR ehrs OR emr OR emrs) OR AB=(ehr OR ehrs OR emr OR emrs)
2	TS=((("computeri?ed" OR "electronic" OR "personal" OR personali?ed) NEAR/1 ("health* data*" OR "health* record*" OR "medical record*" OR "patient record*" OR "pharmaceutical record*")))
3	TS=((system* NEAR/1 ("clinical decision" OR "clinical information" OR "decision support" OR "health information" OR "medical information")))
4	TS=("cds system*" OR "medical record linkage*" OR "patient* portal*" OR "personal health information")
5	#1 OR #2 OR #3 OR #4
6	TS=(client$ OR hospitali* OR inpatient* OR institution* OR outpatient* OR patient*)
7	TS=((("clinical" OR health* OR "medical" OR "nurse" OR "nursing") NEAR/2 (organi?ation* OR "personnel" OR professional* OR provider* OR "staff")))
8	TS=(allergist$ OR anesthesiologist$ OR anaesthesiologist$ OR cardiologist$ OR dermatologist$ OR endocrinologist$ OR gastroenterologist$ OR "general practitioner$" OR geriatrician$ OR midwife* OR midwive* OR neonatologist$ OR nephrologist$ OR neurologist$ OR nurse* OR oncologist$ OR ophthalmologist$ OR otolaryngologist$ OR pathologist$ OR pediatrician$ OR paediatrician$ OR pharmacist* OR physiatrist$ OR physician$ OR "physical therapist$" OR physiotherapist$ OR "physio-therapist$" OR psychotherapist$ OR "psycho-therapist$" OR pulmonologist$ OR radiologist$ OR rheumatologist$ OR surgeon$ OR urologist$)
9	#6 OR #7 OR #8
10	TS=((("artificial" OR comput* OR "machine") NEAR/1 "intelligence"))
11	TS=((("data" OR "information") NEAR/2 (administ* OR custod* OR "dissemination" OR "distribution" OR extract* OR govern* OR link* OR manage* OR retrieval* OR source* OR sharing* OR steward* OR storage*)))
12	TS=(("learning" NEAR/2 ("deep" OR "hierarchical" OR "labeled data" OR "machine" OR "semi-supervised" OR "transfer")))
13	TS=(((machine$ OR network$) NEAR/1 "support vector"))
14	TS=((cloud* OR "knowledge acquisition" OR "knowledge representation$" OR "natural language process*"))
15	#10 OR #11 OR #12 OR #13 OR #14
16	TS=("anonymi?ation" OR blockchain* OR "block chain*" OR "civil suit*" OR confidential* OR "co-own*" OR coown* OR cybersecurit* OR "de-identification$" OR deidentification$ OR "electronic signature*" OR encrypt* OR "jurisprudence" OR law* OR "legal" OR legislat* OR ownership* OR "privileged communication" OR regulation* OR "secrecy" OR secur*)
17	TS=((("computer" OR "cyber" OR "data") NEAR/2 (breach* OR "compromising" OR hacker$ OR "virus" OR "viruses" OR worm*)))
18	TS=((("data" OR "information") NEAR/2 ("masking" OR "protection")))
19	TS=(((patient* OR "property") NEAR/1 right*))
20	TS=((("patient" OR "data") NEAR/1 "privacy"))

21	#16 OR #17 OR #18 OR #19 OR #20
22	#5 AND #9 AND #15 AND #21

5. Cinahl

Interface: Ebsco<br><br>Date of Search: 20 April 2022<br><br>Number of hits: 1,090	Field labels<br><br>• MH+ = exploded Cinahl Heading<br>• MH = non exploded Cinahl Heading<br>• TI = title<br>• AB = abstract<br>• Nx = within x words, regardless of order<br>• * = truncation of word for alternate endings

#	Query
S44	S11 AND S18 AND S32 AND S43
S43	S33 OR S34 OR S35 OR S36 OR S37 OR S38 OR S39 OR S40 OR S41 OR S42
S42	TI (((patient OR data) N1 privacy)) OR AB (((patient OR data) N1 privacy))
S41	TI (((patient* OR property) N1 right*)) OR AB (((patient* OR property) N1 right*))
S40	TI (((data OR information) N2 (masking OR protection))) OR AB (((data OR information) N2 (masking OR protection)))
S39	TI (((computer OR cyber OR data) N2 (breach* OR compromising OR hacker# OR virus OR viruses OR worm*))) OR AB (((computer OR cyber OR data) N2 (breach* OR compromising OR hacker# OR virus OR viruses OR worm*)))
S38	TI ((anonymi?ation OR blockchain* OR "block chain*" OR "civil suit*" OR confidential* OR "co-own*" OR coown* OR cybersecurit* OR "de-identification#" OR deidentification# OR "electronic signature*" OR encrypt* OR jurisprudence OR law* OR legal OR legislat* OR ownership* OR "privileged communication" OR regulation* OR secrecy OR secur*)) OR AB ((anonymi?ation OR blockchain* OR "block chain*" OR "civil suit*" OR confidential* OR "co-own*" OR coown* OR cybersecurit* OR "de-identification#" OR deidentification# OR "electronic signature*" OR encrypt* OR jurisprudence OR law* OR legal OR legislat* OR ownership* OR "privileged communication" OR regulation* OR secrecy OR secur*))
S37	MW "LJ"
S36	MH "Patient Rights"
S35	MH "Patient Access to Records"
S34	MH "Privacy and Confidentiality"
S33	MH "Data Security+"
S32	S19 OR S20 OR S21 OR S22 OR S23 OR S24 OR S25 OR S26 OR S27 OR S28 OR S29 OR S30 OR S31
S31	TI ((cloud* OR "knowledge acquisition" OR "knowledge representation#" OR "natural language process*")) OR AB ((cloud* OR "knowledge acquisition" OR "knowledge representation#" OR "natural language process*"))
S30	TI (((machine# OR network#) N1 "support vector")) OR AB (((machine# OR network#) N1 "support vector"))
S29	TI ((learning N2 (deep OR hierarchical OR "label#ed data" OR machine OR "semi-supervised" OR transfer))) OR AB ((learning N2 (deep OR hierarchical OR "label#ed data" OR machine OR "semi-supervised" OR transfer)))
S28	TI (((data OR information) N2 (administ* OR custod* OR dissemination OR distribution OR extract* OR govern* OR link* OR manage* OR retrieval* OR source* OR sharing* OR steward* OR storage*))) OR AB (((data OR information) N2 (administ* OR custod* OR dissemination OR distribution OR extract* OR govern* OR link* OR manage* OR retrieval* OR source* OR sharing* OR steward* OR storage*)))

S27	TI ((computer N1 (reasoning OR "vision system"))) OR AB ((computer N1 (reasoning OR "vision system")))
S26	TI (((artificial OR comput* OR machine) N1 intelligence)) OR AB (((artificial OR comput* OR machine) N1 intelligence))
S25	MH "Natural Language Processing"
S24	MH "Machine Learning+"
S23	MH "Information Retrieval"
S22	MH "Information Storage"
S21	MH "Data Management"
S20	MH "Cloud Computing+"
S19	MH "Artificial Intelligence"
S18	S12 OR S13 OR S14 OR S15 OR S16 OR S17
S17	TI ((allergist# OR anesthesiologist# OR anaesthesiologist# OR cardiologist# OR dermatologist# OR endocrinologist# OR gastroenterologist# OR "general practitioner#" OR geriatrician# OR midwife* OR midwive* OR neonatologist# OR nephrologist# OR neurologist# OR nurse* OR oncologist# OR ophthalmologist# OR otolaryngologist# OR pathologist# OR paediatrician# OR pediatrician# OR pharmacist* OR physiatrist# OR physician# OR "physical therapist#" OR physiotherapist# OR "physio-therapist#" OR psychotherapist# OR "psycho-therapist#" OR pulmonologist# OR radiologist# OR rheumatologist# OR surgeon# OR urologist#)) OR AB ((allergist# OR anesthesiologist# OR anaesthesiologist# OR cardiologist# OR dermatologist# OR endocrinologist# OR gastroenterologist# OR "general practitioner#" OR geriatrician# OR midwife* OR midwive* OR neonatologist# OR nephrologist# OR neurologist# OR nurse* OR oncologist# OR ophthalmologist# OR otolaryngologist# OR pathologist# OR paediatrician# OR pediatrician# OR pharmacist* OR physiatrist# OR physician# OR "physical therapist#" OR physiotherapist# OR "physio-therapist#" OR psychotherapist# OR "psycho-therapist#" OR pulmonologist# OR radiologist# OR rheumatologist# OR surgeon# OR urologist#))
S16	TI (((clinical OR health* OR medical OR nurse OR nursing) N2 (organi?ation* OR personnel OR professional* OR provider* OR staff))) OR AB (((clinical OR health* OR medical OR nurse OR nursing) N2 (organi?ation* OR personnel OR professional* OR provider* OR staff)))
S15	TI ((client# OR hospitali* OR inpatient* OR institution* OR outpatient* OR patient*)) OR AB ((client# OR hospitali* OR inpatient* OR institution* OR outpatient* OR patient*))
S14	MH "Patients+"
S13	MH "Consumer Participation"
S12	MH "Health Personnel+"
S11	S1 OR S2 OR S3 OR S4 OR S5 OR S6 OR S7 OR S8 OR S9 OR S10
S10	TI (("cds system*" OR "medical record linkage*" OR "patient* portal*" OR "personal health information")) OR AB (("cds system*" OR "medical record linkage*" OR "patient* portal*" OR "personal health information"))
S9	TI ((system* N1 ("clinical decision" OR "clinical information" OR "decision support" OR "health information" OR "medical information"))) OR AB ((system* N1 ("clinical decision" OR "clinical information" OR "decision support" OR "health information" OR "medical information")))
S8	TI (((computeri?ed OR electronic OR personal OR personali?ed) N1 ("health* data*" OR "health* record*" OR "medical record*" OR "patient record*" OR "pharmaceutical record*"))) OR AB (((computeri?ed OR electronic OR personal OR personali?ed) N1 ("health* data*" OR "health* record*" OR "medical record*" OR "patient record*" OR "pharmaceutical record*")))
S7	TI ((ehr OR ehrs OR emr OR emrs)) OR AB ((ehr OR ehrs OR emr OR emrs))

S6	MH "Medical Record Linkage"
S5	MH "Medical Records, Personal"
S4	MH "Health Information Systems"
S3	MH "Electronic Health Records+"
S2	MH "Decision Support Systems, Management"
S1	MH "Decision Support Systems, Clinical"

6. Scopus

Interface: Elsevier<br><br>Date of Search: 20 April 2022<br><br>Number of hits: 5,798<br><br>Comment:	Field labels<br><br>• TITLE-ABS-KEY = title, abstract and author keywords<br>• W/x = within x words, regardless of order<br>• = truncation of word for alternate endings

1	(TITLE-ABS ({ehr} OR {ehrs} OR {emr} OR {emrs} OR "cds system*" OR "medical record linkage*" OR "patient* portal*" OR "personal health information")) OR (TITLE-ABS-KEY ((computeri*ed OR electronic OR personal OR personali*ed) W/1 ("health* data*" OR "health* record*" OR "medical record*" OR "patient record*" OR "pharmaceutical record*"))) OR (TITLE-ABS-KEY (system* W/1 ("clinical decision" OR "clinical information" OR "decision support" OR "health information" OR "medical information")))
2	(TITLE-ABS-KEY (client* OR hospitali* OR inpatient* OR institution* OR outpatient* OR patient*)) OR (TITLE-ABS-KEY ((clinical OR health* OR medical OR nurse OR nursing) W/2 (organization* OR personnel OR professional* OR provider* OR staff))) OR (TITLE-ABS-KEY (allergist* OR an*esthesiologist* OR cardiologist* OR dermatologist* OR endocrinologist* OR gastroenterologist* OR "general practitioner*" OR geriatrician* OR midwife* OR midwive* OR neonatologist* OR nephrologist* OR neurologist* OR nurse* OR oncologist* OR ophthalmologist* OR otolaryngologist* OR pathologist* OR p*ediatrician* OR pharmacist* OR physiatrist* OR physician* OR "physical therapist*" OR physiotherapist* OR "physio-therapist*" OR psychotherapist* OR "psycho-therapist*" OR pulmonologist* OR radiologist* OR rheumatologist* OR surgeon* OR urologist*))
3	(TITLE-ABS-KEY ((artificial OR comput* OR machine) W/1 intelligence)) OR (TITLE-ABS-KEY ((reasoning OR "vision system") W/1 computer)) OR (TITLE-ABS-KEY ((data OR information) W/2 (administ* OR custod* OR dissemination OR distribution OR extract* OR govern* OR link* OR manage* OR retrieval* OR source* OR sharing* OR steward* OR storage*))) OR (TITLE-ABS-KEY (learning W/2 (deep OR hierarchical OR "label*ed data" OR machine OR semi-supervised OR transfer))) OR (TITLE-ABS-KEY ((machine* OR network*) W/1 "support vector")) OR (TITLE-ABS-KEY (cloud* OR "knowledge acquisition" OR "knowledge representation*" OR "natural language process*"))
4	(TITLE-ABS-KEY (anonymi*ation OR blockchain* OR "block chain*" OR "civil suit*" OR confidential* OR co-own* OR coown* OR cybersecurit* OR de-identification* OR deidentification* OR "electronic signature*" OR encrypt* OR jurisprudence OR law* OR legal OR legislat* OR ownership* OR "privileged communication" OR regulation* OR secrecy OR secur*)) OR (TITLE-ABS-KEY ((computer OR cyber OR data) W/2 (breach* OR compromising OR hacker* OR virus OR viruses OR worm*))) OR (TITLE-ABS-KEY ((data OR information) W/2 (masking OR protection))) OR (TITLE-ABS-KEY ((patient* OR property) W/2 right*)) OR (TITLE-ABS-KEY ((patient OR data) W/2 privacy))
5	((TITLE-ABS ({ehr} OR {ehrs} OR {emr} OR {emrs} OR "cds system*" OR "medical record linkage*" OR "patient* portal*" OR "personal health information")) OR (TITLE-ABS-KEY ((computeri*ed OR electronic OR personal OR personali*ed) W/1 ("health* data*" OR "health* record*" OR "medical record*" OR "patient record*" OR "pharmaceutical record*"))) OR (TITLE-ABS-KEY (system* W/1 ("clinical decision" OR "clinical information" OR "decision support" OR "health information" OR "medical information")))) AND ((TITLE-ABS-KEY (client* OR hospitali* OR inpatient* OR institution* OR outpatient* OR patient*)) OR (TITLE-ABS-KEY ((clinical OR health* OR medical OR nurse OR nursing) W/2 (organization* OR personnel OR professional* OR provider* OR staff))) OR (TITLE-ABS-KEY (allergist* OR an*esthesiologist* OR cardiologist* OR dermatologist* OR endocrinologist* OR gastroenterologist* OR "general practitioner*" OR geriatrician* OR midwife* OR midwive* OR neonatologist* OR nephrologist* OR neurologist* OR nurse* OR

	oncologist* OR ophthalmologist* OR otolaryngologist* OR pathologist* OR p*ediatrician* OR pharmacist* OR physiatrist* OR physician* OR "physical therapist*" OR physiotherapist* OR "physio-therapist*" OR psychotherapist* OR "psycho-therapist*" OR pulmonologist* OR radiologist* OR rheumatologist* OR surgeon* OR urologist*))) AND ((TITLE-ABS-KEY ((artificial OR comput* OR machine) W/1 intelligence)) OR (TITLE-ABS-KEY ((reasoning OR "vision system") W/1 computer)) OR (TITLE-ABS-KEY ((data OR information) W/2 (administ* OR custod* OR dissemination OR distribution OR extract* OR govern* OR link* OR manage* OR retrieval* OR source* OR sharing* OR steward* OR storage*))) OR (TITLE-ABS-KEY (learning W/2 (deep OR hierarchical OR "label*ed data" OR machine OR semi-supervised OR transfer))) OR (TITLE-ABS-KEY ((machine* OR network*) W/1 "support vector")) OR (TITLE-ABS-KEY (cloud* OR "knowledge acquisition" OR "knowledge representation*" OR "natural language process*"))) AND ((TITLE-ABS-KEY (anonymi*ation OR blockchain* OR "block chain*" OR "civil suit*" OR confidential* OR co-own* OR coown* OR cybersecurit* OR de-identification* OR deidentification* OR "electronic signature*" OR encrypt* OR jurisprudence OR law* OR legal OR legislat* OR ownership* OR "privileged communication" OR regulation* OR secrecy OR secur*)) OR (TITLE-ABS-KEY ((computer OR cyber OR data) W/2 (breach* OR compromising OR hacker* OR virus OR viruses OR worm*))) OR (TITLE-ABS-KEY ((data OR information) W/2 (masking OR protection))) OR (TITLE-ABS-KEY ((patient* OR property) W/2 right*)) OR (TITLE-ABS-KEY ((patient OR data) W/2 privacy)))

6. Engineering Village (Platform Elsevier)

Interface: Elsevier<br><br>Date of Search: 7 April 2022<br><br>Number of hits: 510<br><br>Comment: Here you need to add which databases were searched	Field labels<br><br>• WN KY = Subject/Title/Abstract<br>• W/x = within x words, regardless of order<br>• = truncation of word for alternate endings

(((((((anonymi?ation OR blockchain* OR {block chain*} OR {civil suit*} OR confidential* OR {co-own*} OR coown* OR cybersecurit* OR {de-identification*} OR deidentification* OR {electronic signature*} OR encrypt* OR jurisprudence OR law* OR legal OR legislat* OR ownership* OR {privileged communication} OR regulation* OR secrecy OR secur*) WN KY)) AND (1884-2022 WN YR)) OR (((((computer OR cyber OR data) AND breach*) WN KY)) AND (1884-2022 WN YR)) OR ((((compromising OR hacker* OR virus OR viruses OR worm*) WN KY)) AND (1884-2022 WN YR)) OR (((((data OR information) AND masking OR protection) WN KY)) AND (1884-2022 WN YR)) OR (((((patient* OR property) AND right*) WN KY)) AND (1884-2022 WN YR)) OR (((((patient OR data) AND privacy) WN KY)) AND (1884-2022 WN YR)))) AND (((((((artificial OR comput* OR machine) AND intelligence) WN KY)) AND (1884-2022 WN YR)) OR (((((reasoning OR {vision system}) AND computer) WN KY)) AND (1884-2022 WN YR)) OR (((((data OR information) AND (administ* OR custod* OR dissemination OR distribution OR extract* OR govern* OR link* OR manage* OR retrieval* OR source* OR sharing* OR steward* OR storage*)) WN KY)) AND (1884-2022 WN YR)) OR (((((deep OR hierarchical OR {label*ed data} OR machine OR {semi-supervised} OR transfer) AND learning) WN KY)) AND (1884-2022 WN YR)) OR (((({support vector} AND (machine* OR network*)) WN KY)) AND (1884-2022 WN YR)) OR ((((cloud* OR {knowledge acquisition} OR {knowledge representation*} OR {natural language process*}) WN KY)) AND (1884-2022 WN YR)))) AND ((((((client* OR hospitali* OR inpatient* OR {in-patient*} OR institution* OR outpatient* OR {out-patient} OR patient*) WN KY)) AND (1884-2022 WN YR)) AND (((((clinical OR health* OR medical OR nurse OR nursing) AND (organi*ation* OR personnel OR professional* OR provider* OR staff)) WN KY)) AND (1884-2022 WN YR)) AND ((((allergist* OR an*esthesiologist* OR cardiologist* OR dermatologist* OR endocrinologist* OR gastroenterologist* OR {general practitioner*} OR geriatrician* OR midwife* OR neonatologist* OR nephrologist* OR neurologist* OR nurse* OR oncologist* OR ophthalmologist* OR otolaryngologist* OR pathologist* OR p*ediatrician* OR pharmacist* OR psychiatrist* OR physician* OR {physical therapist*} OR physiotherapist* OR psychotherapist* OR {psychotherapist*} OR pulmonologist* OR radiologist* OR rheumatologist* OR surgeon* OR urologist*) WN KY)) AND (1884-2022 WN YR)))) AND ((((((ehr OR ehrs OR emr OR emrs OR {cds system*} OR {medical record linkage*} OR {patient* portal*} OR {personal health information}) WN KY)) AND (1884-2022 WN YR)) OR (((((computeri?ed OR electronic OR personal OR personali?ed) AND ({health* data*} OR {health* record*} OR {medical record*} OR {patient record*} OR {pharmaceutical record*})) WN KY)) AND (1884-2022 WN YR)) OR ((((system AND ({clinical decision} OR {clinical information} OR {decision support} OR {health information} OR {medical information})) WN KY)) AND (1884-2022 WN YR)))))

S2; GPOC Supplementary Meta-Analysis Forest Plots

Policies Prop Meta

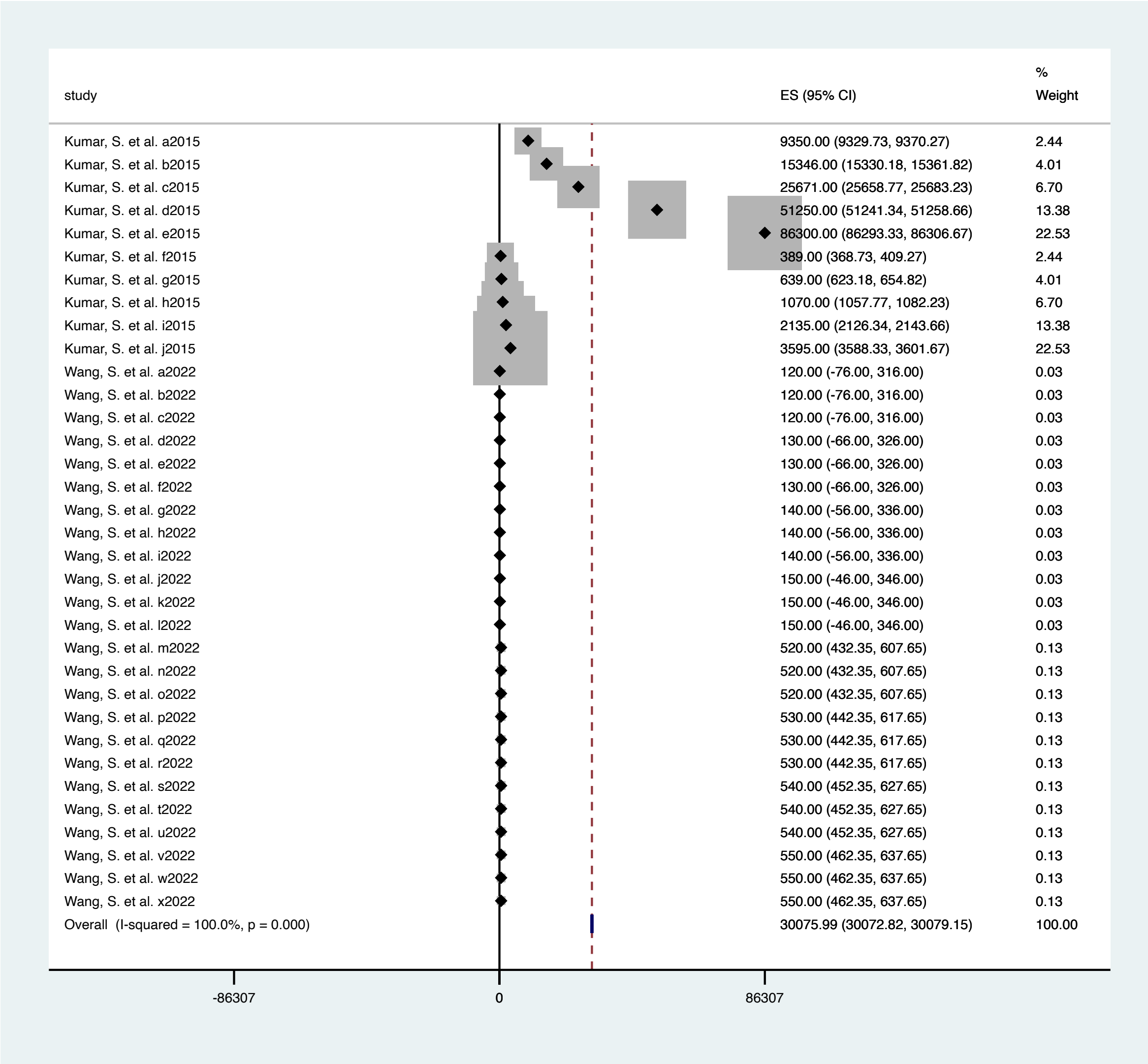

Policies Prop Meta log

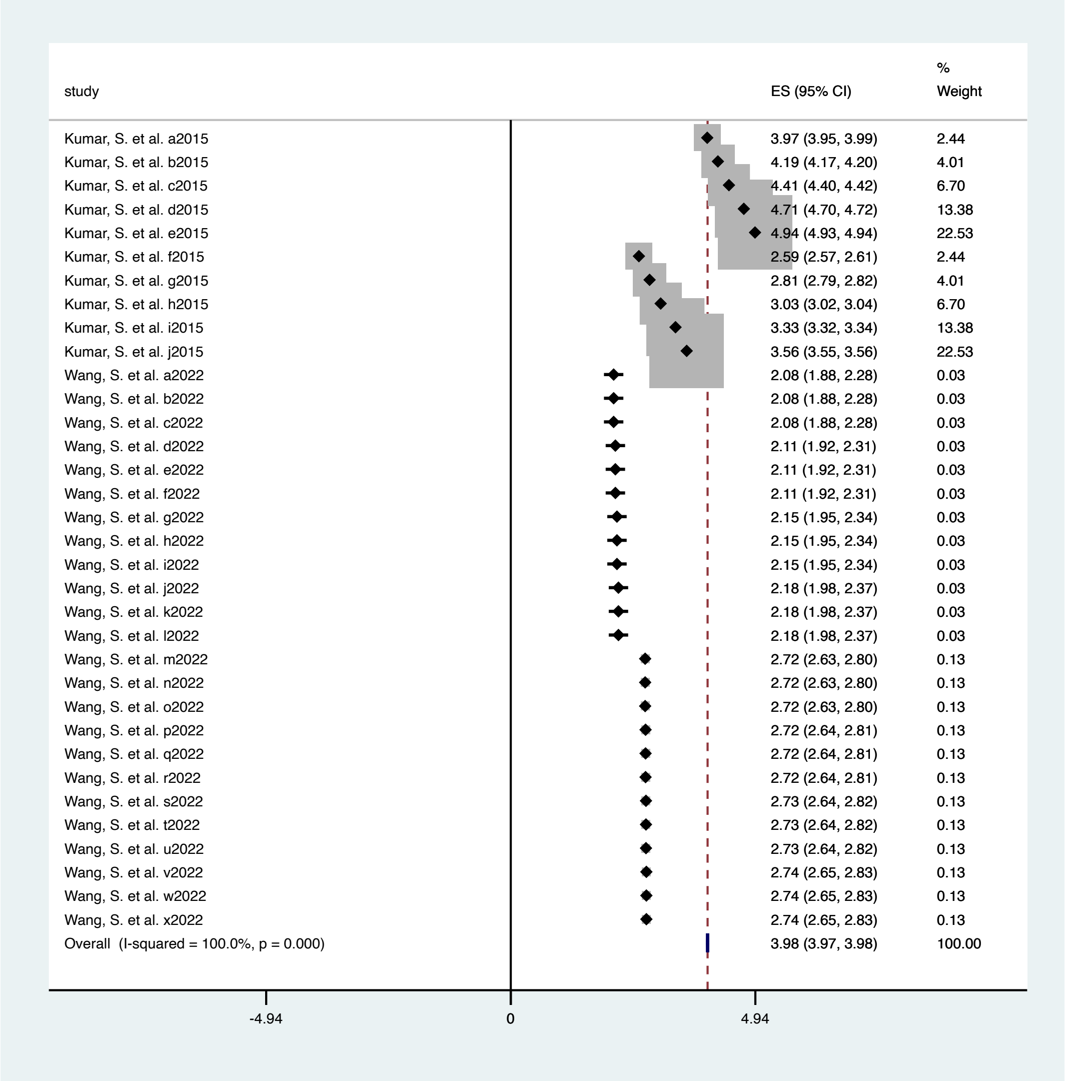

Run Time Prop Meta

study	n	runt	runt_log	sd	_LCI	_UCI	_WT	weight~n
Kocabas, O. et al. a2015	7	6.5	.812913	377964	-740790.3	740803.3	.0082446	12874.07
Kocabas, O. et al. b2015	11	9.4	.973128	301511	-590942	590960.8	.0129558	12874.07
Kocabas, O. et al. c2015	13	28.5	1.45484	277350	-543567.7	543624.7	.0153114	12874.07
Kocabas, O. et al. d2015	16	34.9	1.54283	250000	-489956.1	490025.9	.0188448	12874.07
Kocabas, O. et al. e2015	18	40.3	1.60531	235702	-461927.7	462008.3	.0212004	12874.07

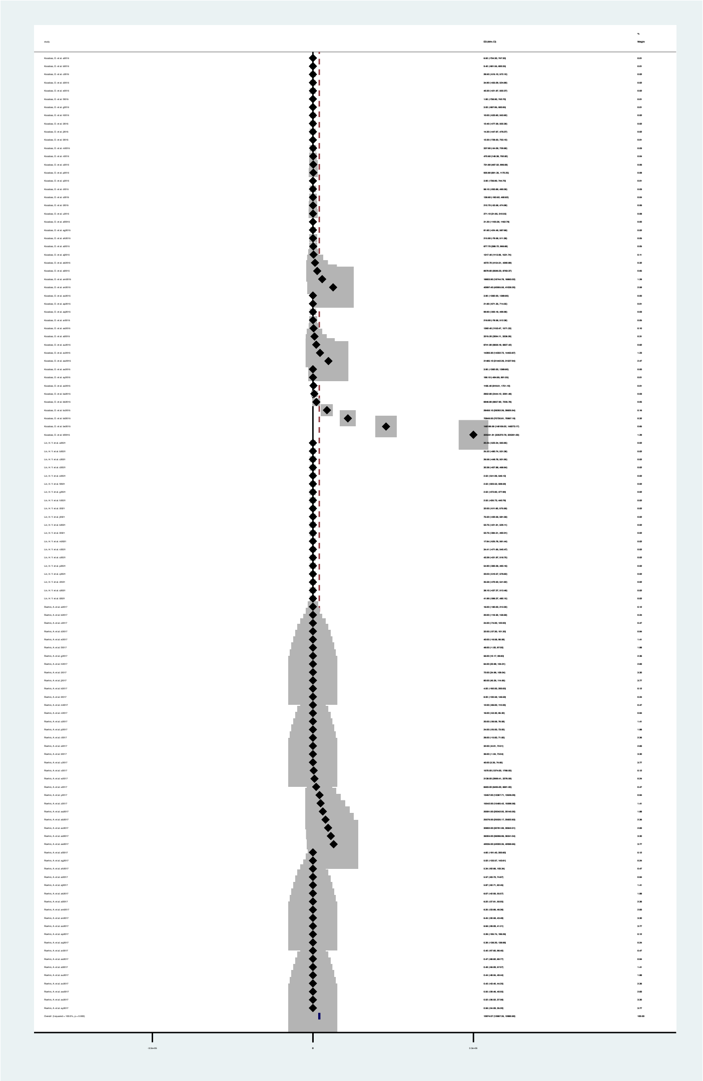

Run Time Prop Meta log

study	n	runt	runt_log	sd	weight~n	_LCI	_UCI	_WT
**Kocabas, O. et al. a2015**	**7**	**6.5**	**.812913**	**377964**	**12874.07**	**-740795.9**	**740797.6**	**.0082446**

weight~b
**1.975848**

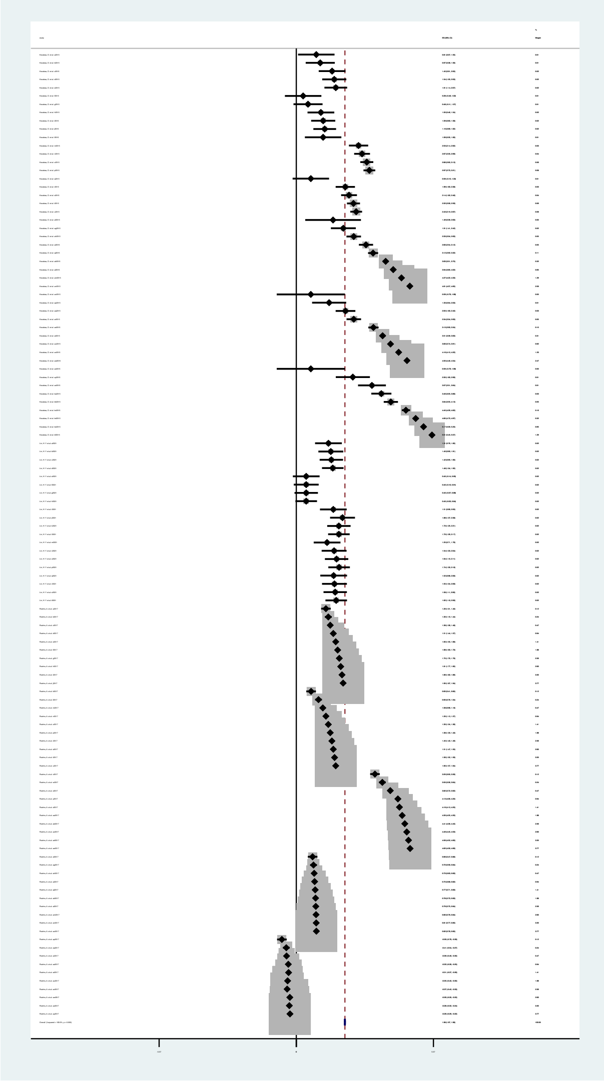

Encryption Time Prop Meta

```
---------------------+--------------------------------------------------
I-V pooled ES        | 80.759      80.755    80.764         100.00
---------------------+--------------------------------------------------
```

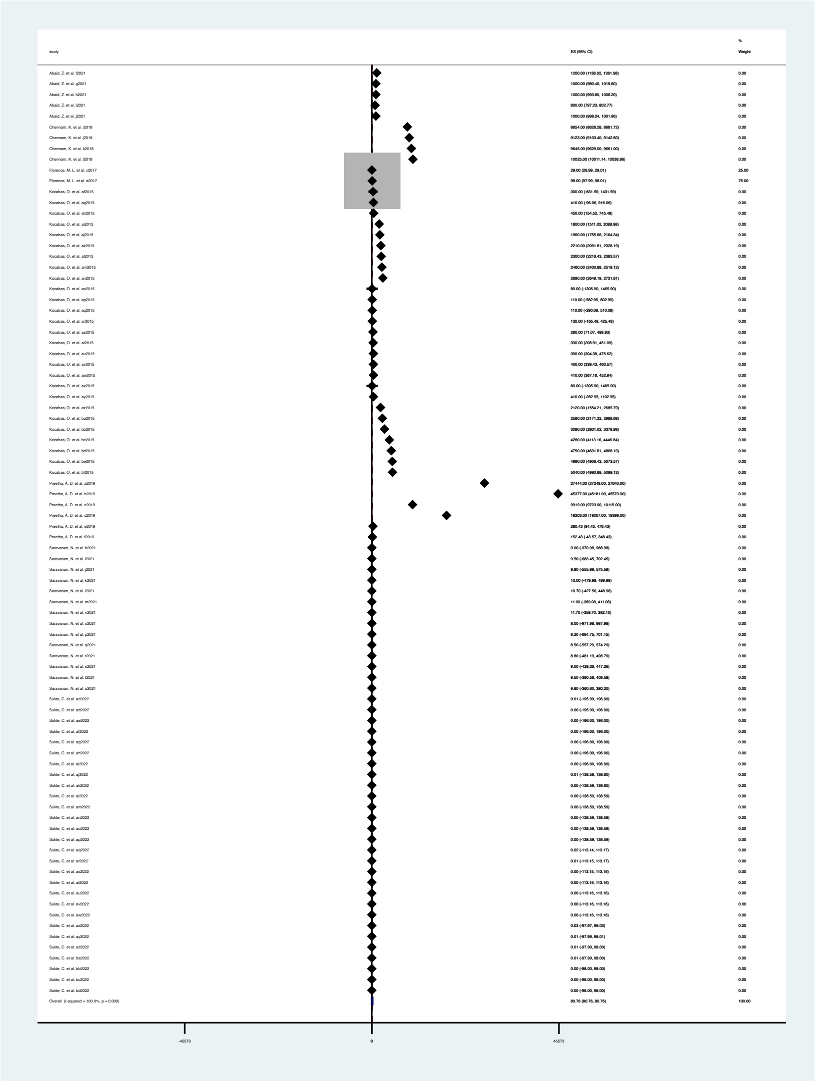

Encryption Time Prop Meta
log

```
-----------------------+-----------------------------------------------------
I-V pooled ES          |  1.859        1.859       1.859         100.00
-----------------------+-----------------------------------------------------
```

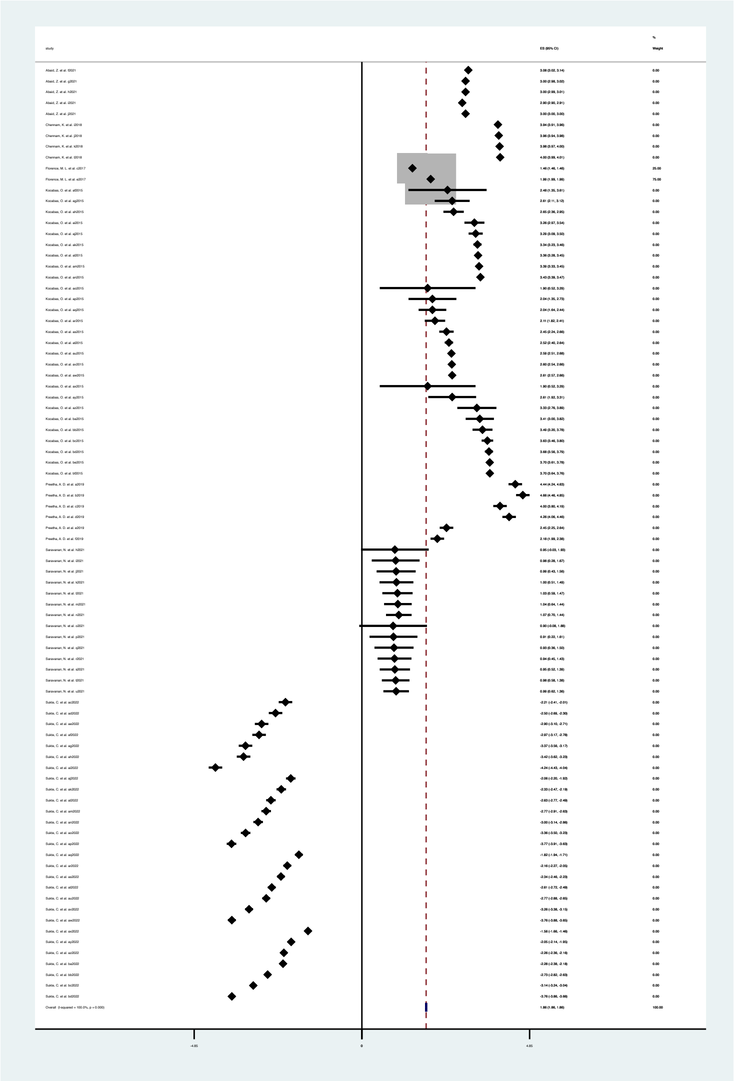

Decryption Time Prop Meta

```
[illegible]
---------------------+------------------------------------------------
I-V pooled ES        | 59.502      59.497    59.506        100.00
---------------------+------------------------------------------------

  Heterogeneity chi-squared =  1.3e+08 (d.f. = 71) p = 0.000
  I-squared (variation in ES attributable to heterogeneity) = 100.0%

  Test of ES=0 : z= 26609.98 p = 0.000
```

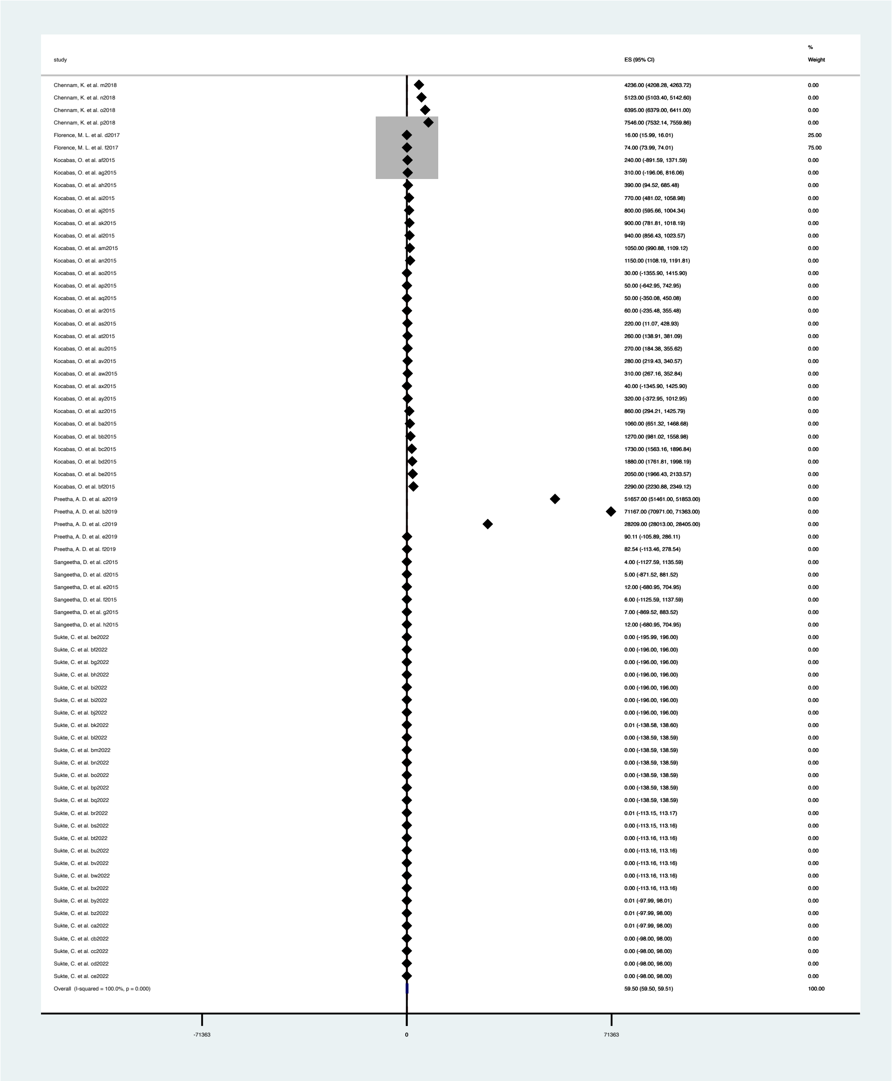

Decryption Time Prop Meta
log

```
---------------------+--------------------------------------------------
I-V pooled ES        |  1.703       1.703     1.703        100.00
---------------------+--------------------------------------------------

  Heterogeneity chi-squared =  1.7e+10 (d.f. = 71) p = 0.000
  I-squared (variation in ES attributable to heterogeneity) = 100.0%

  Test of ES=0 : z=  7.6e+05 p = 0.000
```

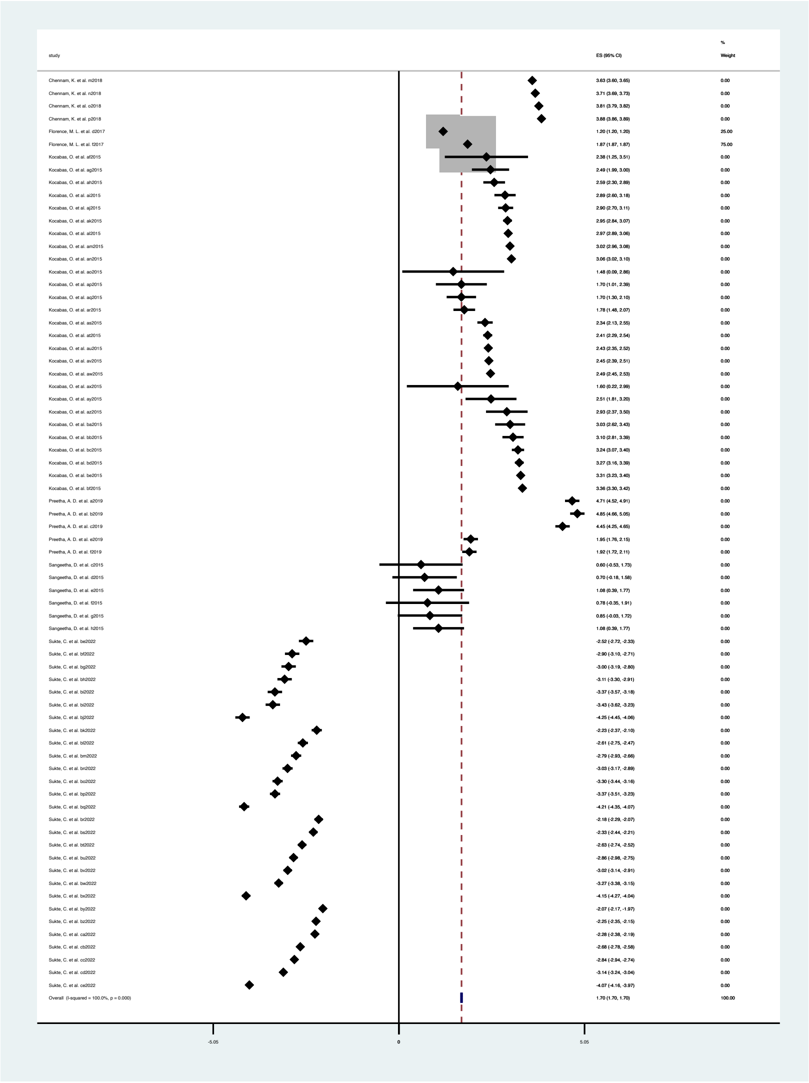

Time Analysis Prop Meta

```
---------------------+---------------------------------------------------
I-V pooled ES        |3951.640    3948.637   3954.642      100.00
---------------------+---------------------------------------------------

  Heterogeneity chi-squared =  3.6e+07 (d.f. = 25) p = 0.000
  I-squared (variation in ES attributable to heterogeneity) = 100.0%

  Test of ES=0 : z= 2579.77 p = 0.000
```

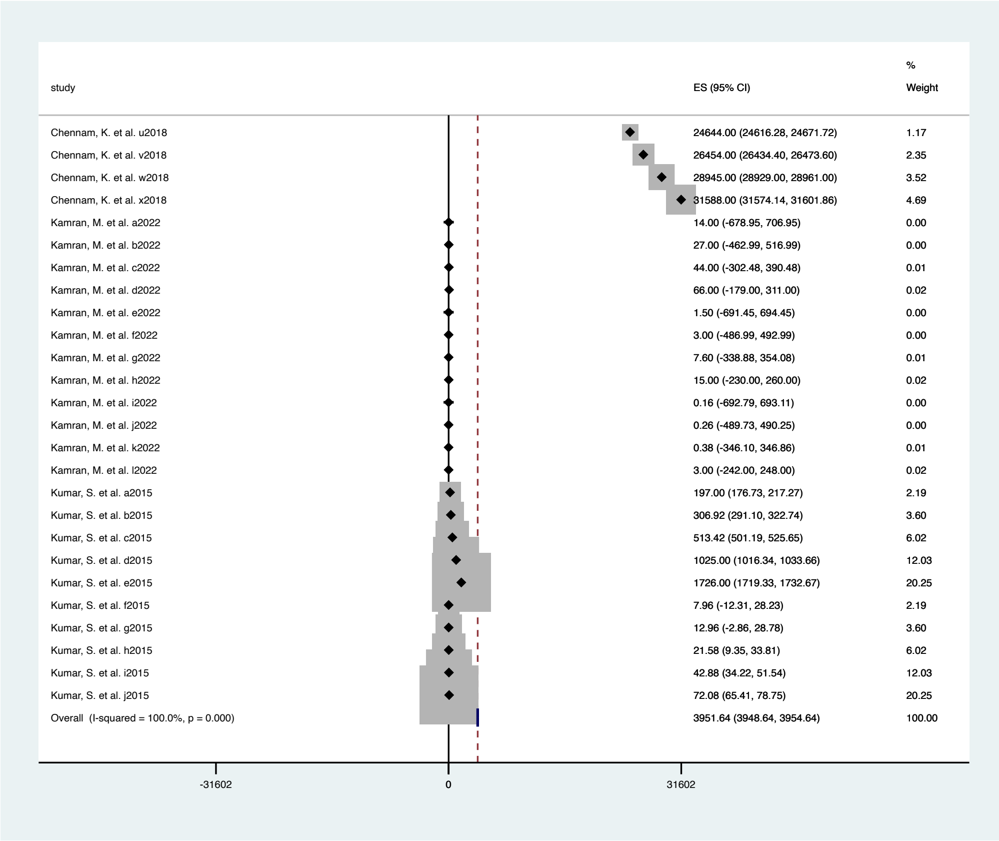

Time Analysis Prop Meta

```
---------------------+----------------------------------------------------
I-V pooled ES        |  2.558       2.555     2.561        100.00
---------------------+----------------------------------------------------

  Heterogeneity chi-squared =  4.2e+05 (d.f. = 25) p = 0.000
  I-squared (variation in ES attributable to heterogeneity) = 100.0%

  Test of ES=0 : z= 1669.63 p = 0.000
```

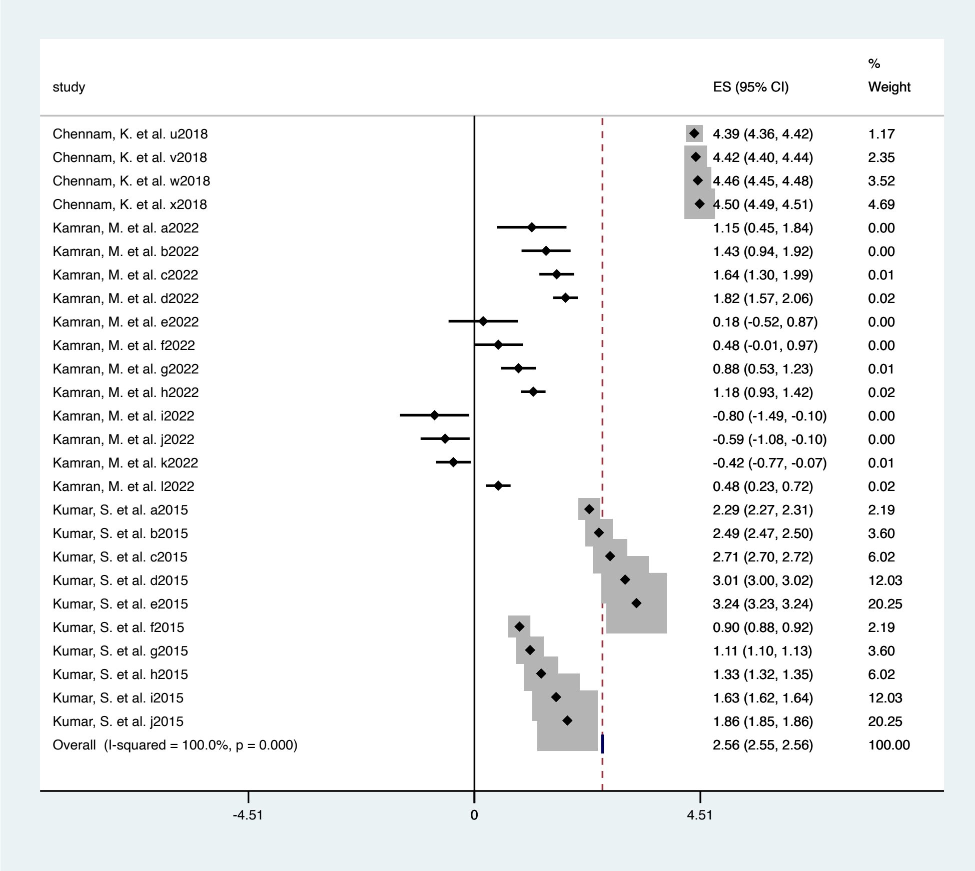

Gas Analysis Prop Meta

```
---------------------+--------------------------------------------------
I-V pooled ES        | 7.0e+04     7.0e+04    7.0e+04      100.00
---------------------+--------------------------------------------------

  Heterogeneity chi-squared =  1.5e+06 (d.f. = 7) p = 0.000
  I-squared (variation in ES attributable to heterogeneity) = 100.0%

  Test of ES=0 : z= 1727.94 p = 0.000
```

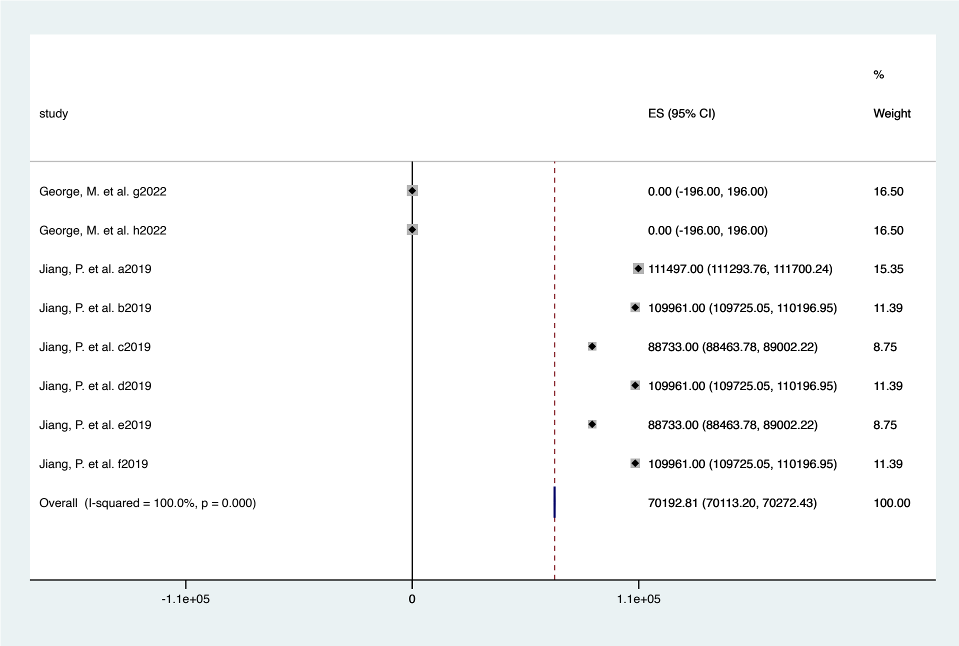

Gas Analysis Prop Meta log

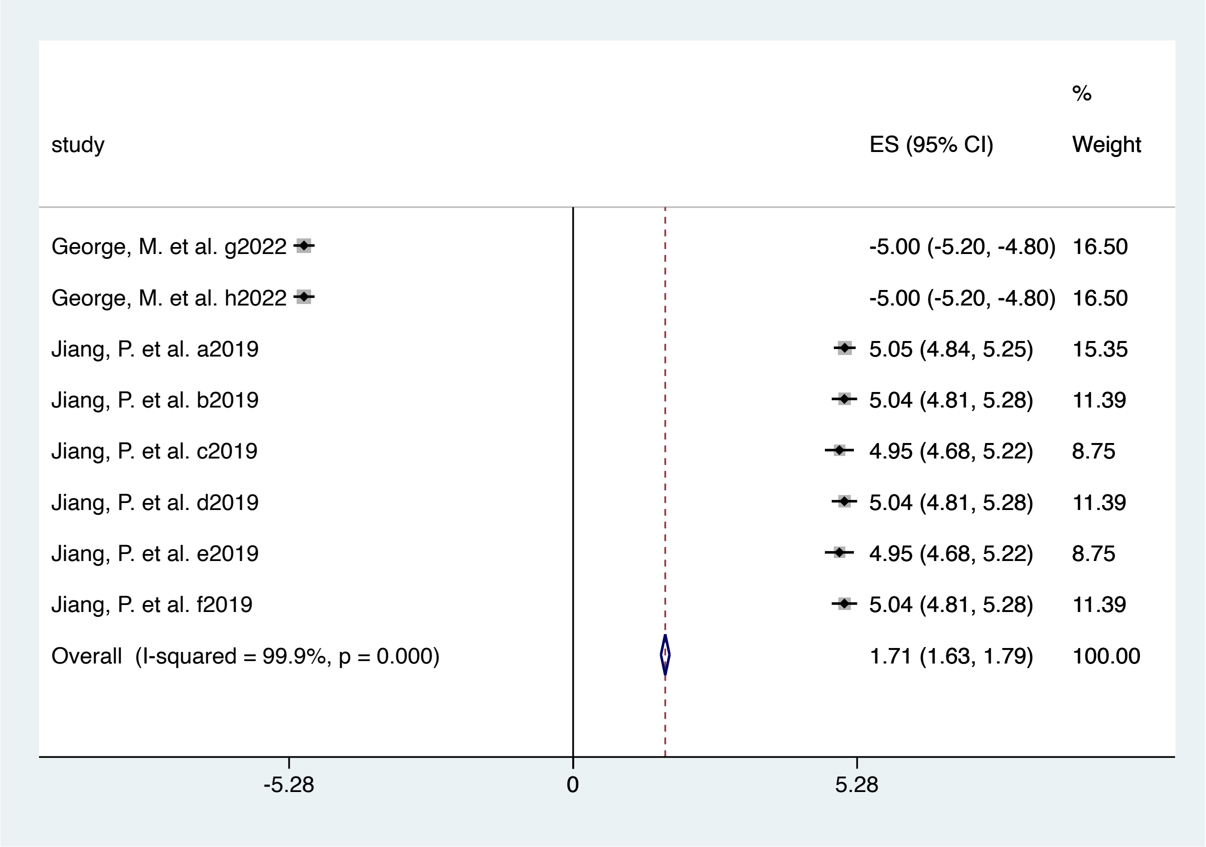

KeyGen Time Prop Meta

```
---------------------+---------------------------------------------------
I-V pooled ES        |143.118     121.014   165.223        100.00
---------------------+---------------------------------------------------

  Heterogeneity chi-squared = 2429.59 (d.f. = 45) p = 0.000
  I-squared (variation in ES attributable to heterogeneity) =  98.1%

  Test of ES=0 : z=  12.69 p = 0.000
```

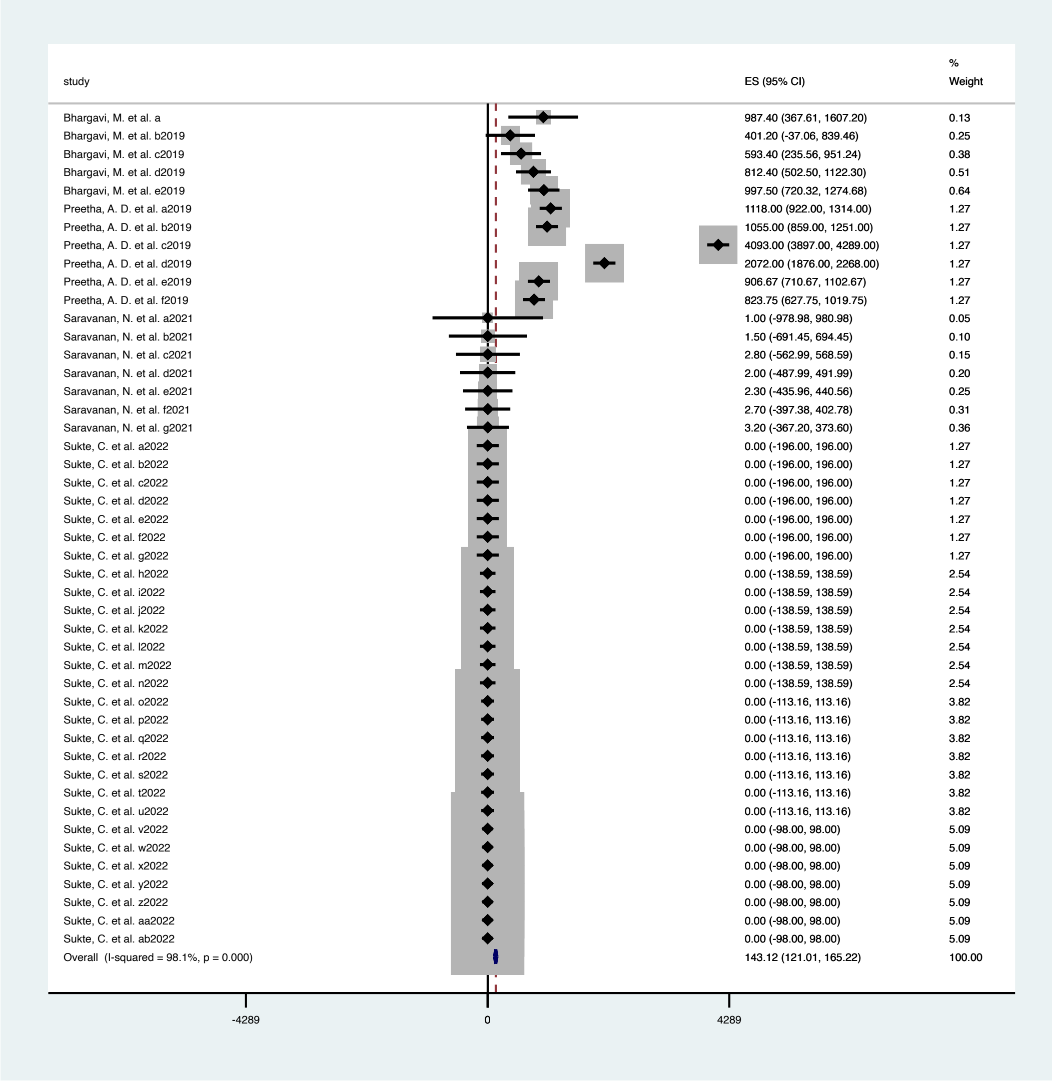

KeyGen Time Prop Meta log

```
---------------------+--------------------------------------------------
I-V pooled ES        | -4.493      -4.515     -4.471        100.00
---------------------+--------------------------------------------------

  Heterogeneity chi-squared = 52411.78 (d.f. = 45) p = 0.000
  I-squared (variation in ES attributable to heterogeneity) =  99.9%

  Test of ES=0 : z= 398.41 p = 0.000
```

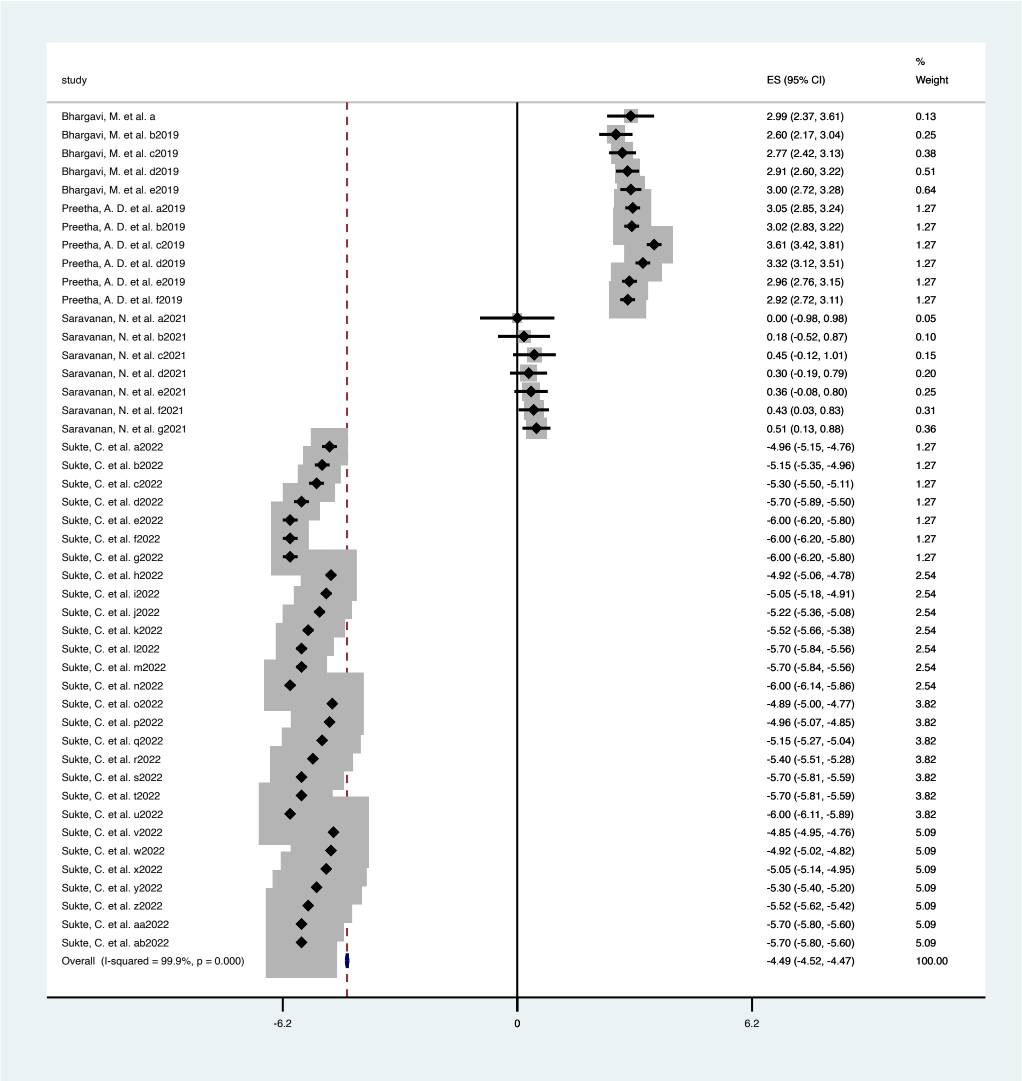

Ratio of Means Encryption Meta i

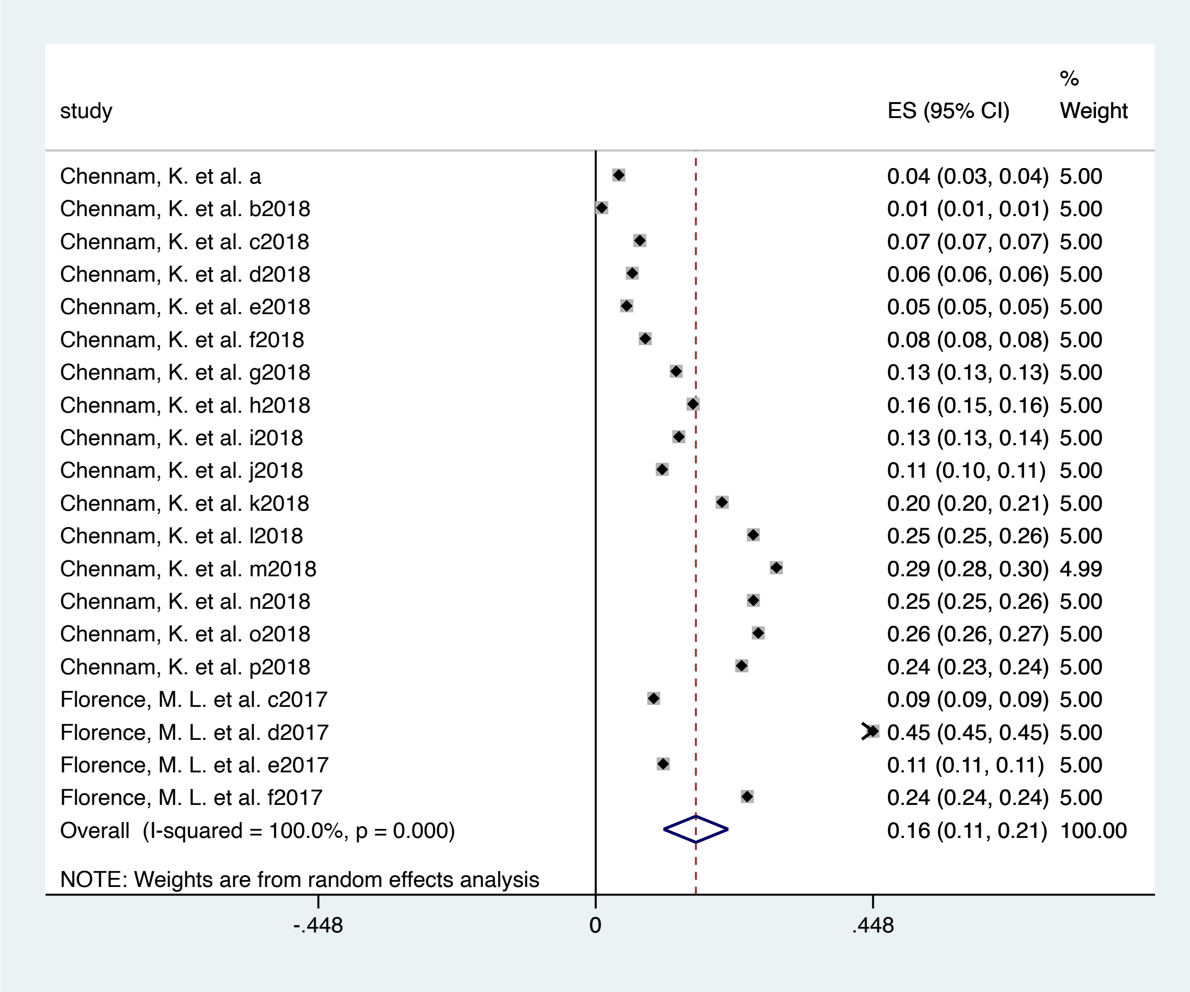

Ratio of Means Encryption Meta i

Study	ES	[95% Conf.	Interval]	% Weight
Chennam, K. et al. a	**0.037**	**0.034**	**0.041**	**5.00**
Chennam, K. et al. b	**0.010**	**0.009**	**0.011**	**5.00**
Chennam, K. et al. c	**0.072**	**0.069**	**0.074**	**5.00**
Chennam, K. et al. d	**0.059**	**0.057**	**0.062**	**5.00**
Chennam, K. et al. e	**0.050**	**0.046**	**0.054**	**5.00**
Chennam, K. et al. f	**0.080**	**0.076**	**0.084**	**5.00**
Chennam, K. et al. g	**0.130**	**0.126**	**0.134**	**5.00**
Chennam, K. et al. h	**0.157**	**0.154**	**0.161**	**5.00**
Chennam, K. et al. i	**0.135**	**0.128**	**0.141**	**5.00**
Chennam, K. et al. j	**0.108**	**0.103**	**0.112**	**5.00**
Chennam, K. et al. k	**0.204**	**0.200**	**0.209**	**5.00**
Chennam, K. et al. l	**0.255**	**0.250**	**0.259**	**5.00**
Chennam, K. et al. m	**0.292**	**0.283**	**0.301**	**4.99**
Chennam, K. et al. n	**0.255**	**0.249**	**0.261**	**5.00**
Chennam, K. et al. o	**0.263**	**0.258**	**0.268**	**5.00**
Chennam, K. et al. p	**0.236**	**0.232**	**0.240**	**5.00**
Florence, M. L. et a	**0.094**	**0.094**	**0.094**	**5.00**
Florence, M. L. et a	**0.448**	**0.448**	**0.448**	**5.00**
Florence, M. L. et a	**0.109**	**0.109**	**0.109**	**5.00**
Florence, M. L. et a	**0.245**	**0.245**	**0.245**	**5.00**
D+L pooled ES	**0.162**	**0.110**	**0.214**	**100.00**

Heterogeneity chi-squared = **5.8e+10** (d.f. = **19**) p = **0.000**
I-squared (variation in ES attributable to heterogeneity) = **100.0%**
Estimate of between-study variance Tau-squared = **0.0142**

Test of ES=0 : z= **6.07** p = **0.000**

Ratio of Means Encryption Meta c

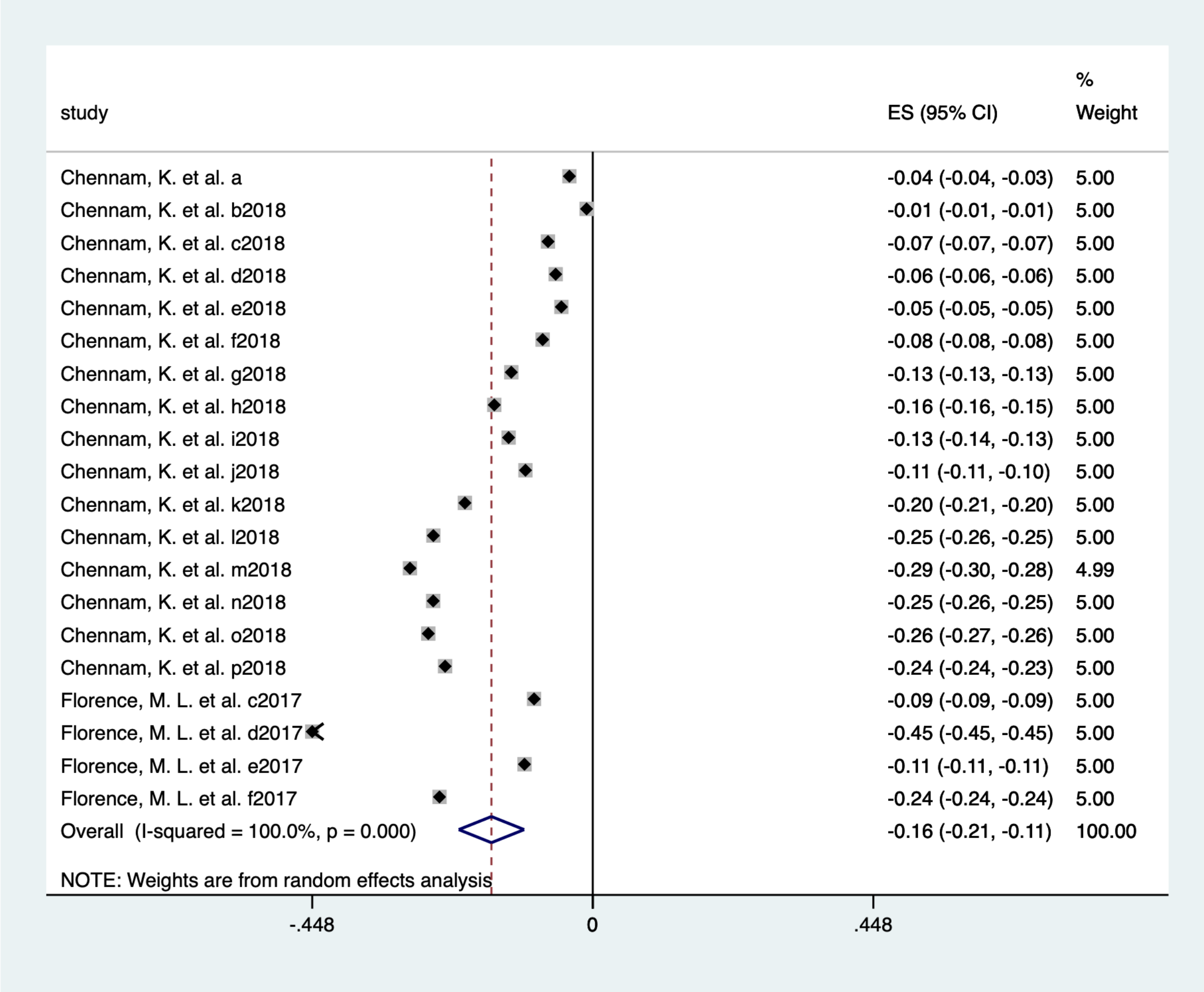

Ratio of Means Encryption Meta c

Study	ES	[95% Conf. Interval]		% Weight
Chennam, K. et al. a	**-0.037**	**-0.041**	**-0.034**	**5.00**
Chennam, K. et al. b	**-0.010**	**-0.011**	**-0.009**	**5.00**
Chennam, K. et al. c	**-0.072**	**-0.074**	**-0.069**	**5.00**
Chennam, K. et al. d	**-0.059**	**-0.062**	**-0.057**	**5.00**
Chennam, K. et al. e	**-0.050**	**-0.054**	**-0.046**	**5.00**
Chennam, K. et al. f	**-0.080**	**-0.084**	**-0.076**	**5.00**
Chennam, K. et al. g	**-0.130**	**-0.134**	**-0.126**	**5.00**
Chennam, K. et al. h	**-0.157**	**-0.161**	**-0.154**	**5.00**
Chennam, K. et al. i	**-0.135**	**-0.141**	**-0.128**	**5.00**
Chennam, K. et al. j	**-0.108**	**-0.112**	**-0.103**	**5.00**
Chennam, K. et al. k	**-0.204**	**-0.209**	**-0.200**	**5.00**
Chennam, K. et al. l	**-0.255**	**-0.259**	**-0.250**	**5.00**
Chennam, K. et al. m	**-0.292**	**-0.301**	**-0.283**	**4.99**
Chennam, K. et al. n	**-0.255**	**-0.261**	**-0.249**	**5.00**
Chennam, K. et al. o	**-0.263**	**-0.268**	**-0.258**	**5.00**
Chennam, K. et al. p	**-0.236**	**-0.240**	**-0.232**	**5.00**
Florence, M. L. et a	**-0.094**	**-0.094**	**-0.094**	**5.00**
Florence, M. L. et a	**-0.448**	**-0.448**	**-0.448**	**5.00**
Florence, M. L. et a	**-0.109**	**-0.109**	**-0.109**	**5.00**
Florence, M. L. et a	**-0.245**	**-0.245**	**-0.245**	**5.00**
D+L pooled ES	**-0.162**	**-0.214**	**-0.110**	**100.00**

Heterogeneity chi-squared = **5.8e+10** (d.f. = **19**) p = **0.000**
I-squared (variation in ES attributable to heterogeneity) = **100.0%**
Estimate of between-study variance Tau-squared = **0.0142**

Test of ES=0 : z= **6.07** p = **0.000**

S3; PRISMA 2020 Checklist GPOC

Section and Topic	Item #	Checklist item	Location where item is reported
**TITLE**			
Title	1	Identify the report as a systematic review.	Article, page 2
**ABSTRACT**			
Abstract	2	See the PRISMA 2020 for Abstracts checklist.	Article, page 2-3
**INTRODUCTION**			
Rationale	3	Describe the rationale for the review in the context of existing knowledge.	Article, page 2, and 3-4
Objectives	4	Provide an explicit statement of the objective(s) or question(s) the review addresses.	Article, page 2, and 3-4
**METHODS**			
Eligibility criteria	5	Specify the inclusion and exclusion criteria for the review and how studies were grouped for the syntheses.	Supplementary Files – Meta Folder – Search Documentation Strategy, and article page 5
Information sources	6	Specify all databases, registers, websites, organisations, reference lists and other sources searched or consulted to identify studies. Specify the date when each source was last searched or consulted.	Supplementary Files – Meta Folder – Search Documentation Strategy, and article page 5
Search strategy	7	Present the full search strategies for all databases, registers and websites, including any filters and limits used.	Supplementary Files – Meta Folder – Search Documentation Strategy, and article page 5
Selection process	8	Specify the methods used to decide whether a study met the inclusion criteria of the review, including how many reviewers screened each record and each report retrieved, whether they worked independently, and if applicable, details of automation tools used in the process.	Article, page 5-6
Data collection process	9	Specify the methods used to collect data from reports, including how many reviewers collected data from each report, whether they worked independently, any processes for obtaining or confirming data from study investigators, and if applicable, details of automation tools used in the process.	Article, page 5
Data items	10a	List and define all outcomes for which data were sought. Specify whether all results that were compatible with each outcome domain in each study were sought (e.g. for all measures, time points, analyses), and if not, the methods used to decide which results to collect.	Article, page 5-7
	10b	List and define all other variables for which data were sought (e.g. participant and intervention characteristics, funding sources). Describe any assumptions made about any missing or unclear information.	Article, page 5-6, and page 25
Study risk of bias assessment	11	Specify the methods used to assess risk of bias in the included studies, including details of the tool(s) used, how many reviewers assessed each study and whether they worked independently, and if applicable, details of automation tools used in the process.	The ROBINS-I and the RobVis tool, page 6, and figure 4 on page 13
Effect measures	12	Specify for each outcome the effect measure(s) (e.g. risk ratio, mean difference) used in the synthesis or presentation of results.	Article, page 10
Synthesis	13a	Describe the processes used to decide which	Article, page 5-6

Section and Topic	Item #	Checklist item	Location where item is reported
methods		studies were eligible for each synthesis (e.g. tabulating the study intervention characteristics and comparing against the planned groups for each synthesis (item #5)).	
	13b	Describe any methods required to prepare the data for presentation or synthesis, such as handling of missing summary statistics, or data conversions.	Article, page 5-6
	13c	Describe any methods used to tabulate or visually display results of individual studies and syntheses.	Article, page 5-6
	13d	Describe any methods used to synthesize results and provide a rationale for the choice(s). If meta-analysis was performed, describe the model(s), method(s) to identify the presence and extent of statistical heterogeneity, and software package(s) used.	Article, page 5-6
	13e	Describe any methods used to explore possible causes of heterogeneity among study results (e.g. subgroup analysis, meta-regression).	Foest plot, figure 3 on page 9
	13f	Describe any sensitivity analyses conducted to assess robustness of the synthesized results.	Article, page 5-6
Reporting bias assessment	14	Describe any methods used to assess risk of bias due to missing results in a synthesis (arising from reporting biases).	ROB, Page 6 and 12-13
Certainty assessment	15	Describe any methods used to assess certainty (or confidence) in the body of evidence for an outcome.	Page 10-13
**RESULTS**			
Study selection	16a	Describe the results of the search and selection process, from the number of records identified in the search to the number of studies included in the review, ideally using a flow diagram.	Page 7
	16b	Cite studies that might appear to meet the inclusion criteria, but which were excluded, and explain why they were excluded.	Page 7
Study characteristics	17	Cite each included study and present its characteristics.	Page 7-9, and Supplementary Files – Meta Folder – GPOC Meta Supplementary
Risk of bias in studies	18	Present assessments of risk of bias for each included study.	ROB, Page 6 and 12-13
Results of individual studies	19	For all outcomes, present, for each study: (a) summary statistics for each group (where appropriate) and (b) an effect estimate and its precision (e.g. confidence/credible interval), ideally using structured tables or plots.	Page 7-9, and Supplementary Files – Meta Folder – GPOC Meta Supplementary
Results of syntheses	20a	For each synthesis, briefly summarise the characteristics and risk of bias among contributing studies.	ROB, Page 6 and 12-13
	20b	Present results of all statistical syntheses conducted. If meta-analysis was done, present for each the summary estimate and its precision (e.g. confidence/credible interval) and measures of statistical heterogeneity. If comparing groups, describe the direction of the effect.	Page 7-9, and Supplementary Files – Meta Folder – GPOC Meta Supplementary
	20c	Present results of all investigations of possible causes of heterogeneity among study results.	Page 7-13
	20d	Present results of all sensitivity analyses conducted to assess the robustness of the synthesized results.	Page 7-13
Reporting biases	21	Present assessments of risk of bias due to missing	Page 7-13

Section and Topic	Item #	Checklist item	Location where item is reported
		results (arising from reporting biases) for each synthesis assessed.	
Certainty of evidence	22	Present assessments of certainty (or confidence) in the body of evidence for each outcome assessed.	Page 7-13
**DISCUSSION**			
Discussion	23a	Provide a general interpretation of the results in the context of other evidence.	Page 14-22
	23b	Discuss any limitations of the evidence included in the review.	Page 14-22
	23c	Discuss any limitations of the review processes used.	Page 14-22
	23d	Discuss implications of the results for practice, policy, and future research.	Page 14-22
**OTHER INFORMATION**			
Registration and protocol	24a	Provide registration information for the review, including register name and registration number, or state that the review was not registered.	Page 2
	24b	Indicate where the review protocol can be accessed, or state that a protocol was not prepared.	Supplementary Files – Meta Folder – Search Documentation Strategy, and article page 5
	24c	Describe and explain any amendments to information provided at registration or in the protocol.	Supplementary Files – Meta Folder – Search Documentation Strategy, and article page 5
Support	25	Describe sources of financial or non-financial support for the review, and the role of the funders or sponsors in the review.	Page 25
Competing interests	26	Declare any competing interests of review authors.	Page 25
Availability of data, code and other materials	27	Report which of the following are publicly available and where they can be found: template data collection forms; data extracted from included studies; data used for all analyses; analytic code; any other materials used in the review.	Supplementary Files – Meta Folder

From: Page MJ, McKenzie JE, Bossuyt PM, Boutron I, Hoffmann TC, Mulrow CD, et al. The PRISMA 2020 statement: an updated guideline for reporting systematic reviews. BMJ 2021;372:n71. doi: 10.1136/bmj.n71

For more information, visit: http://www.prisma-statement.org/

S4; GPOC Word Cloud

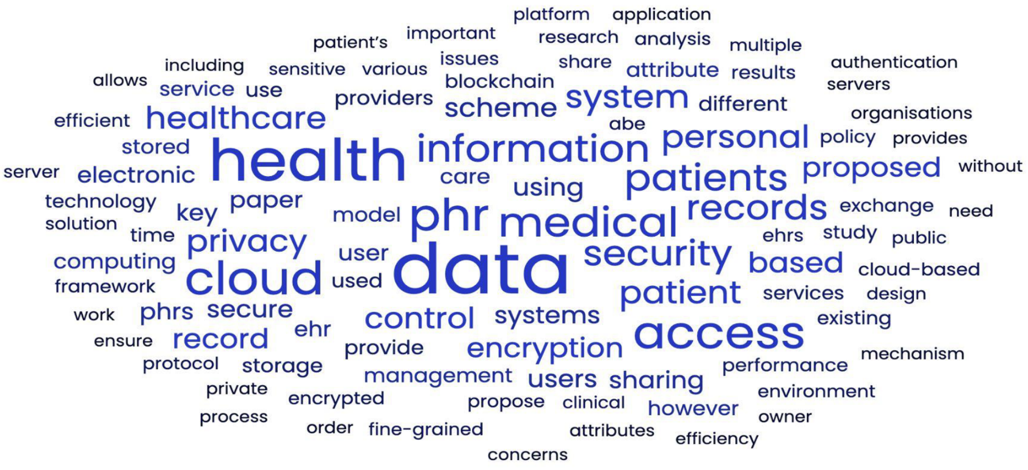

Supplement: Supplementary file 1 — Supplementary Information [file 41467_2024_46503_MOESM1_ESM.pdf]
